# KLK1 as an Epithelial‐Specific Brake Inhibits Colorectal Tumorigenesis by Suppressing B1R‐Mediated Fibroblast Phenotypic Transition

**DOI:** 10.1002/advs.202507063

**Published:** 2025-08-26

**Authors:** Lisha Zhou, Meijing Wang, Shunji Liu, Limei Gu, Shijia Liu, Qianming Du, Tianyi Zhang, Yinuo Ma, Lixin Zhao, Jiaming Wang, Qiang Xu, Tingsheng Ling, Haibo Cheng, Hongqi Chen, Yang Sun

**Affiliations:** ^1^ State Key Laboratory of Pharmaceutical Biotechnology and Nanjing Drum Tower Hospital School of Life Sciences Chemistry and Biomedicine Innovation Center Nanjing University 163 Xianlin Avenue Nanjing Jiangsu 210023 P. R. China; ^2^ Institute of Structural Pharmacology & TCM Chemical Biology College of Pharmacy Fujian University of Traditional Chinese Medicine No. 1, Qiuyang Road Fuzhou Fujian 350122 P. R. China; ^3^ Jiangsu Key Laboratory of New Drug Research and Clinical Pharmacy Xuzhou Medical University Xuzhou 221004 P. R. China; ^4^ Digestive Endoscopy Center Affiliated Hospital of Nanjing University of Chinese Medicine Jiangsu Province Hospital of Chinese Medicine Nanjing 210029 P. R. China; ^5^ The Affiliated Hospital of Nanjing University of Chinese Medicine Jiangsu Province Hospital of Chinese Medicine Nanjing Jiangsu 210029 P. R. China; ^6^ General Clinical Research Center Nanjing First Hospital Nanjing Medical University Nanjing 210006 P. R. China; ^7^ Department of Laboratory Medicine The First Affiliated Hospital of USTC Division of Life Sciences and Medicine University of Science and Technology of China Hefei 230026 P. R. China; ^8^ Jiangsu Collaborative Innovation Center of Traditional Chinese Medicine in Prevention and Treatment of Tumor The First Clinical Medical College Nanjing University of Chinese Medicine 138 Xianlin Avenue Nanjing Jiangsu 210023 P. R. China; ^9^ Department of General Surgery Shanghai Jiao Tong University Affiliated Sixth People's Hospital 600 Yishan Road Shanghai 200233 P. R. China

**Keywords:** bradykinin B1 receptors, cancer‐associated fibroblasts, extracellular matrix, intestinal barrier, tissue kallikrein

## Abstract

Inflammatory bowel disease (IBD) is increasing worldwide, and the persistence of chronic inflammation may lead to colitis‐associated colorectal cancer (CAC). KLK1 expression is reduced in colitis, and its potential role in the intestinal mucosal barrier is still unclear. Here, KLK1 is investigated whether a supplement can reduce colitis and colorectal carcinogenesis. This study investigated KLK1's protective function in intestinal barrier integrity using Dextran Sulfate Sodium Salt (DSS) / Azoxymethane (AOM)‐DSS‐induced colitis/CAC models, Apc‐deficient mice, and human clinical samples. KLK1‐AAV2 knockdown mice exhibited exacerbated colitis symptoms, including severe diarrhea and impaired mucosal barrier markers, while KLK1 levels are notably reduced in ulcerative colitis patients and colorectal cancer specimens. Mechanistically, bradykinin receptor B1 (B1R) upregulation in CAC models activated extracellular matrix pathways, driving fibroblast phenotypic shifts that disrupt stromal homeostasis. Crucially, KLK1 supplementation reversed these pathological changes, demonstrating its dual role in maintaining epithelial barrier function and regulating fibroblast‐ECM interactions. These findings position KLK1 as a potential therapeutic target for colitis and CRC chemoprevention, offering novel insights into IBD pathogenesis through its modulation of mucosal protection and stromal remodeling processes.

## Graphical abstract

KLK1 downregulation disrupts the intestinal mucosal barrier and impairs kallikrein‐kinin signaling, thereby reducing Lys‐des‐Arg^9^‐BK production. This enhances B1R activation on ADAMDEC1⁺ fibroblasts, promoting inflammation and extracellular matrix (ECM) remodeling. The resulting iCAFs promote colorectal cancer progression, highlighting a novel KLK1‐B1R axis linking colitis to tumorigenesis via MAPK signaling.

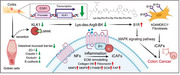

## 1 Introduction

Ulcerative colitis (UC) is a chronic nonspecific inflammatory bowel disease with persistent or recurrent diarrhea, abdominal pain, and mucus, pus, and blood in the stool as the main manifestations.^[^ ^1^ ^]^ Long‐term chronic inflammation will increase the incidence of colitis‐associated colorectal cancer (CAC) in UC patients. The order of onset of colorectal cancer is adenoma‐dysplasia‐cancer, while the order of onset of CAC is inflammation‐dysplasia‐cancer.^[^ ^2^ ^]^ The kallikrein‐kinin system (KKS) is part of the humoral defense system involved in inflammatory responses. However, in severe inflammation, KKS amplifies the inflammatory cascade and leads to tissue destruction and chronic inflammation.

The KLKs are a class of secreted serine proteases with trypsin or chymotrypsin activity, consisting of 15 members (KLK1‐15).^[^ ^3^, ^4^ ^]^ The KLK family is well known for the role of KLK1 in the kallikrein‐kinin system and the clinical applicability of KLK3 as a biomarker for prostate cancer screening.^[^ ^5^ ^]^ Kinin is one of the inflammatory mediators, the most common kinins are bradykinin (BK) and lysyl‐bradykinin (Lys‐BK), also known as kallidin. BK is released from high molecular weight kininogen (HMWK) with the help of plasma kallikrein, while Lys‐BK is released from low molecular weight kininogen (LMWK) by tissue kallikrein.^[^ ^5^, ^6^ ^]^ Both BK and Lys‐BK can bind to B2 receptors to regulate blood pressure, vascular permeability, and inflammatory responses, but Lys‐BK is often converted to BK, so the biological effects of the two are highly similar. The C‐terminal arginine of BK and Lys‐BK can be cleaved by carboxypeptidases N and M to des‐Arg^9^‐BK and Lys‐des‐Arg^9^‐BK, which mainly exert their effects by binding to B1R, especially under pathological conditions such as chronic inflammation and pain.^[^ ^6^ ^]^

Our findings support human translational relevance, with KLK1 levels being reduced in IBD patients with UC and further reduced in patients with colorectal low‐grade endothelial neoplasia and colon cancer, suggesting KLK1 dysregulation in IBD and potential opportunities for intervention using KLK1. In addition, a reduction in KLK1 protein expression was concurrent with a reduction in EGR1 transcript levels, suggesting that EGR1 transcription is inhibited under inflammatory conditions, leading to a reduction in KLK1.

Therefore, we hypothesized that KLK1 plays a protective role in inflammatory bowel diseases (such as ulcerative colitis) by maintaining intestinal epithelial barrier function, and its downregulation may lead to intestinal barrier damage, thereby promoting the development of inflammation and the transformation process of colon cancer. This is the first report demonstrating that KLK1 is involved in maintaining the integrity of the intestinal mucosal barrier and influencing the dynamic balance of fibroblast phenotype.

## 2 Results

### 2.1 Single‐Cell Transcriptome Analysis Revealed that KLK1 Expression was Specifically Downregulated in Epithelial Cells in Ulcerative Colitis

**Figure 1:**
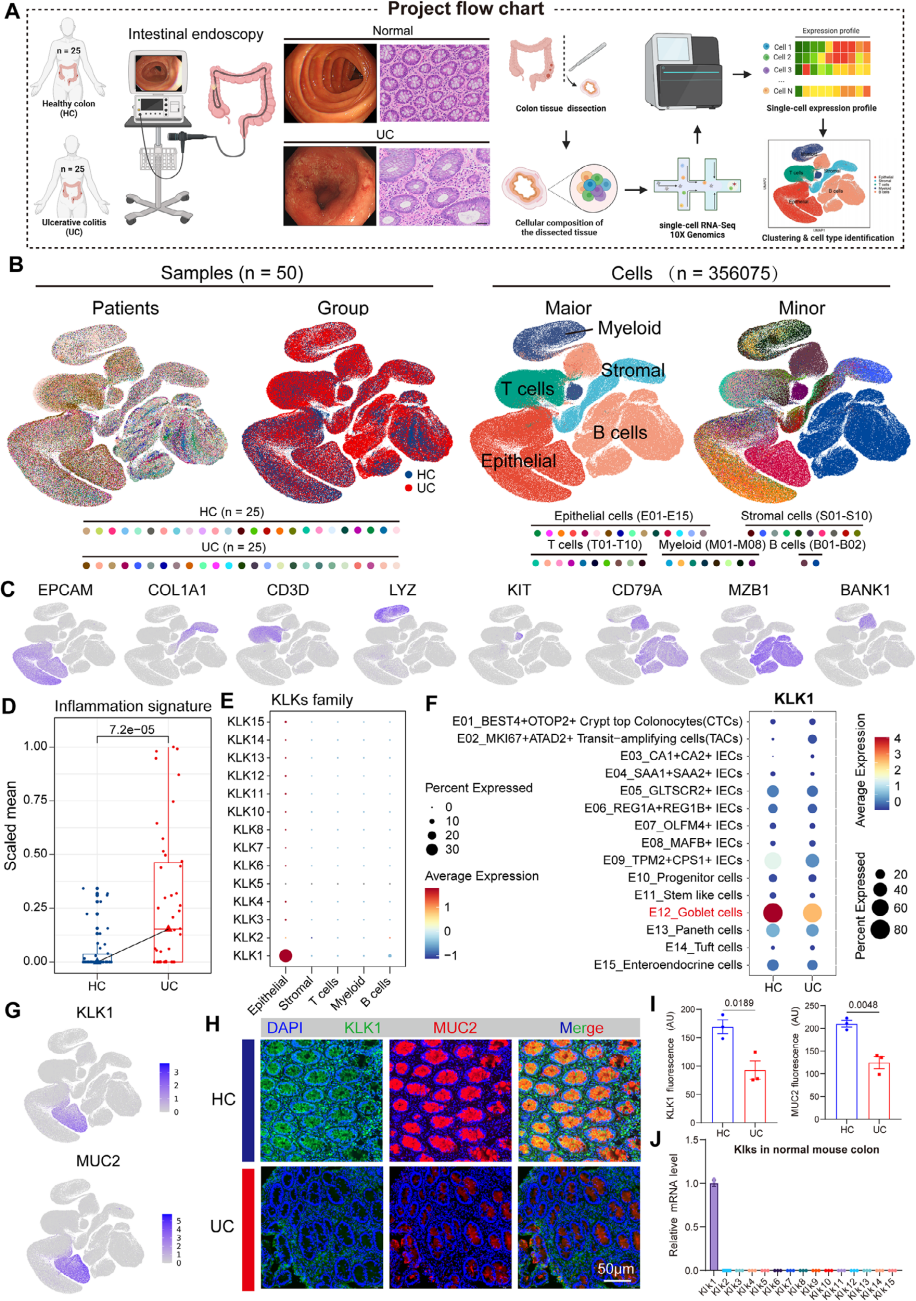
Single‐cell transcriptomic analysis reveals that the expression of KLK1 is downregulated in epithelial cells of human ulcerative colitis tissue. A) The schematics of sample collection, scRNA‐seq transcriptomic analysis of ulcerative colitis, and combined analysis with public datasets. B) Uniform manifold approximation and projection (UMAP) of 356 075 cells analyzed by scRNA‐seq across 50 samples. Data information was placed in Table S1 (Supporting Information). Clusters were annotated by the canonical markers. C) UMAP plot showing the expression levels of the selected markers in all cell subtypes. D) Box plot showing the mean expression of inflammation signatures in cells from different sample groups. The boxes indicate the 25% quantile, median, and 75% quantile; the points indicate the individual signatures. E) The expression levels of the KLKs family genes in different cell subtypes. Dot size indicates the fraction of expressing cells, and the colors represent normalized gene expression levels. F) The expression levels of the KLK1 in epithelial cell subtypes. Dot size indicates the fraction of expressing cells, and the colors represent normalized gene expression levels. G) UMAP plot showing the expression levels of KLK1 and MUC2 in all cells. H,I) Immunofluorescence staining of MUC2^+^ KLK1^+^ cells in human ulcerative colitis and healthy colon with KLK1 (green), MUC2 (red), and DAPI (blue) antibodies. Data are representative of three independent experiments (n=3 per group). J) The relative mRNA expression level of KLK1 between normal colon cells in C57BL/6 mice. Primer sequence information is shown in Table S2 (Supporting Information). Scale bars: 50 µm. All data are shown as the mean ± SEM. Data are representative of three independent experiments. The *P* value was analyzed by one‐way ANOVA with Tukey's multiple comparisons, and all *P* values are marked with specific values in the graph.

In order to explore which molecules in the kallikrein family play a dominant role in colitis progression, we integrated single‐cell transcriptome data from colonic mucosal tissue from 25 UC patients and 25 healthy individuals (**Figure** **1**A). Further, we divided the cells in the colonic mucosal tissue into five subpopulations, namely epithelial cells, stromal cells, T cells, B cells, and myeloid cells by cell subpopulation recognition and dimensionality reduction cluster analysis (Figure 1B,C). As pro‐tumor inflammation is an important marker of cancer,^[^ ^7^ ^]^ we compared the expression patterns of inflammation‐related genes in the UC state (Figure 1D).^[^ ^8^ ^]^ The KLKs family plays an important role in tumor progression and is considered to be a key factor in regulating tumor cell proliferation, migration, and invasion.^[^ ^9^, ^10^ ^]^ KLKs are involved in various activities such as skin inflammation, wound healing, and viral susceptibility. KLK5 and KLK7 were physiological desquamation, which is involved in the development of inflammatory skin diseases with barrier abnormalities and is associated with the skin barrier.^[^ ^11^ ^]^ Next, we attempted to explore whether the KLK family was related to the intestinal mucosal barrier. By analyzing scRNA‐seq data from healthy people and UC patients, KLK1 was most abundant in the KLK family and was significantly enriched in epithelial cells (Figure 1E). Further, KLK1 was expressed low in UC patients and decreased in goblet cell‐specific expression at the site of inflammation, which was validated by immunofluorescence with MUC2 colocalization (Figure 1F–I). In addition, we verified that KLK1 was most expressed in the colon of normal mice by extracting primary intestinal epithelial cells (Figure 1J).

The specific mechanism of KLK1 in the pathogenesis of UC has not been reported. This study takes KLK1 as a starting point to explore the specific role of KLK1 in the occurrence of colitis and the development of colorectal cancer, hoping to find new targets for the intestinal epithelial barrier and the treatment of colitis and colorectal cancer.

### 2.2 KLK1 Deficiency Exacerbates Colitis by Disrupting Intestinal Mucosal Barrier Function

**Figure 2:**
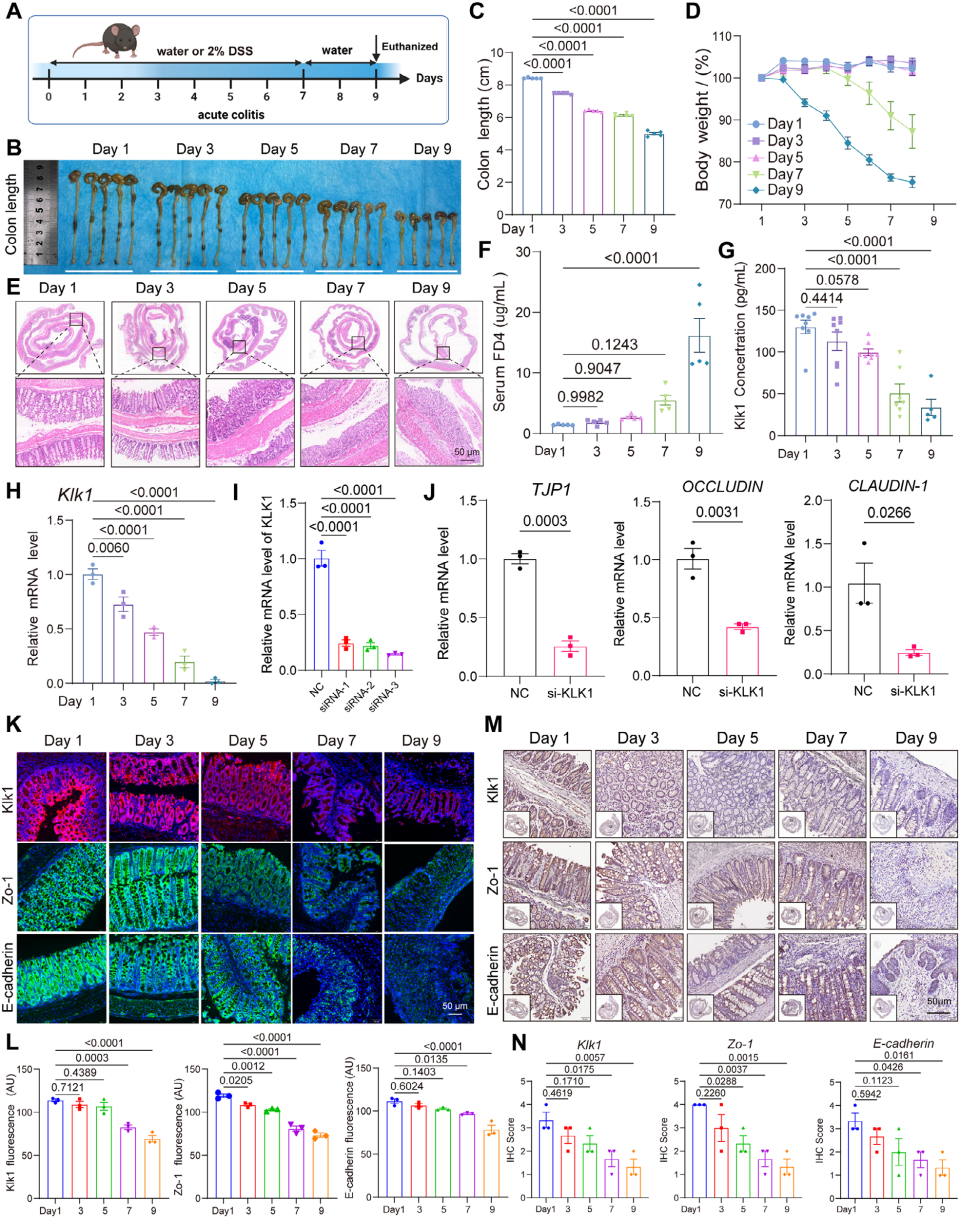
KLK1 is decreased in DSS induced acute colitis model mice. A) Schematic diagram of the experimental design of 2% DSS‐induced acute colitis. B) Representative picture of the colons from different groups (n=5 per group) and C) colon length. D) Body weight curves denote the changes in the mean body weight of mice recorded daily in different groups. E) Photomicrographs show representative images of H&E staining. F) FITC‐Dextran 4 (FD4) was used to detect intestinal permeability in C57BL/6 mice (n=5 per group). G) Detection of mouse serum Klk1 concentration by ELISA (n > 5 per group). H) Relative mRNA level of Klk1 in 2% DSS‐induced acute colitis. All data are expressed as mean ± SEM from three independent experiments. I) Relative mRNA expression after KLK1 knockdown by siRNA. J) Relative mRNA levels of TJP1, OCCLUDIN, and CLAUDIN‐1 after KLK1 knockdown in NCM460. K,L) Immunofluorescence staining of KLK1, Zo‐1 and E‐cadherin in C57BL/6 mice colon with Klk1 (red), Zo‐1 (green), E‐cadherin (green), and DAPI (blue) antibodies (n=3 per group). M,N) Immunohistochemistry of Swiss rolls showing the changes in the expression of KLK1 and intestinal epithelial mucosal barrier markers in an acute colitis model (n=3 per group). Scale bars: 50 µm. All data are shown as the mean ± SEM. Data are representative of three independent experiments. The *P* value was analyzed by one‐way ANOVA with Tukey's multiple comparisons, and all *P* values are marked with specific values in the graph.

To explore the role of KLK1 in the development of colitis, we used a 2% DSS‐induced acute colitis mouse model. Mice drank 2% DSS or water freely for 7 consecutive days, and mice in the DSS group lost 10–20% of their body weight (**Figure** **2**A,D). As the DSS intake time increased, the colon damage of mice worsened, manifested as diarrhea, severe bloody stools, and gradual shortening of the colon (Figure 2B,C). HE staining results showed that mice had varying degrees of colon ulcers on the 5th day, accompanied by mucosal edema, goblet cell loss, crypt swelling and destruction, mucosal and submucosal infiltration, and epithelial cell damage (Figure 2E). We established a FITC‐dextran detection system to evaluate the permeability of the intestinal barrier and found that the level of FITC‐dextran increased with increasing inflammation, indicating increased intestinal permeability (Figure 2F). The expression of KLK1 in mouse serum also gradually decreased with increasing inflammation (Figure 2G). In addition, we extracted RNA from mouse tissues and found that the mRNA level of KLK1 gradually decreased with increasing inflammation (Figure 2H). We knocked down KLK1 in the human colon cell line NCM460 and found that the mRNA levels of mucosal barrier‐related markers such as TJP1, OCCLUDIN, and CLAUDIN‐1 were significantly reduced, indicating that the expression of KLK1 also affected the intestinal mucosal barrier between epithelial cells (Figure 2I,J). Immunofluorescence and immunohistochemistry results showed that the protein expression of KLK1, Zo‐1, and E‐Cadherin in intestinal epithelial tissue decreased with the aggravation of inflammation (Figure 2K–N).

The occurrence of intestinal inflammation leads to a shortening of the length of the colon, and persistent inflammation promotes the development of tumors in the colon area. In daily life, if acute enteritis is left untreated, the condition gradually becomes chronic enteritis, which is a self‐protective response of the body to remove pathogens, remove damaged tissue, and initiate the repair process. Chronic inflammation, on the other hand, is a more persistent inflammatory response, usually due to the failure of acute inflammation to effectively remove pathogens or damaged tissues, or due to recurrent inflammation caused by chronic irritation. Chronic inflammation can persist for weeks, months, or even years. Therefore, we next explored the expression of KLK1 in a DSS‐induced mouse model of chronic colitis. We used a 2% DSS‐induced mouse model of chronic colitis to investigate the role of KLK1 in enteritis. Mice were administered ad libitum drinking water containing 2% DSS for 3 cycles, each cycle maintained for 1 week, and the water was restored for 2 weeks after the end of the first and second cycles, and 3 weeks after the end of the last cycle (Figure S1A,B, Supporting Information). As inflammation continued, colonic damage intensified in mice, showing significant colon shortening (Figure S1C,D, Supporting Information), epithelial hyperplasia, and mucosal fibrosis (Figure S1E,F, Supporting Information). With the increase of DSS cycles, the mRNA level of KLK1 gradually decreased, and its expression in serum also decreased (Figure S1G,H, Supporting Information).

Furthermore, as chronic intestinal inflammation persisted and intensified, the expression of KLK1 and Zo‐1 decreased. Loss of E‐cadherin expression is a hallmark of epithelial‐mesenchymal transition (EMT) and is associated with an increased risk of cancer metastasis. The results showed that the expression of the cell adhesion molecule E‐Cadherin also decreased to varying degrees as inflammation persisted (Figure S1I–L, Supporting Information). These results indicated that the expression of KLK1 was positively correlated with Zo‐1 and E‐Cadherin, suggesting that KLK1 may be involved in the maintenance of colonic mucosal barrier homeostasis and epithelial‐mesenchymal transition in the process of chronic colitis.

The above results indicate that the expression of KLK1 is negatively correlated with the occurrence of colitis, and its decreased expression will affect the imbalance of the intestinal mucosal barrier.

### 2.3 Endogenous KLK1 Deficiency Leads to Intestinal Mucosal Barrier Imbalance and Exacerbates Colitis

**Figure 3:**
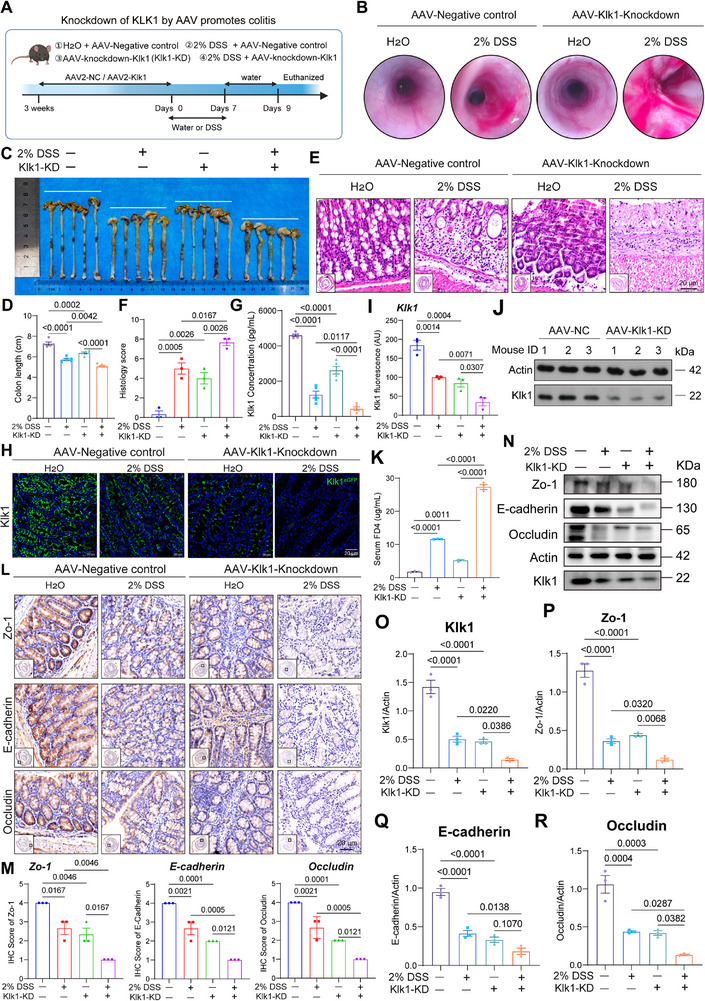
KLK1 reduction by AAV in DSS‐induced inflammation aggravates intestinal mucosal barrier damage. A) Schematic diagram of the experimental design of reducing KLK1 by AAV in the DSS‐induced colitis model. B) High‐resolution endoscopic images of the colon in C57BL/6 mice with AAV‐induced acute colitis treated with H_2_O and 2% DSS, respectively. C) Representative picture of the colons from different groups and D) colon length (n=5 per group). E,F) The representative images of H&E staining and histopathological grading of inflammation in C57BL/6 mice colon with AAV‐induced acute colitis treated with H2O and 2% DSS (n=3 per group). G) Detection of mouse serum Klk1 concentration by ELISA (n=5 per group). H–J) Immunofluorescence showing the protein expression of KLK1, and all data are expressed as mean ± SEM from three independent experiments (n=3 per group). K) FITC‐Dextran 4 (FD4) was used to detect intestinal permeability in C57BL/6 mice (n=3 per group). L,M) IHC staining showing the intestinal mucosal barrier‐related indicators after KLK1 reduction by AAV. Scale bars: 20 µm. (n=3 per group). N–R) Protein levels of Klk1 and intestinal epithelial mucosal barrier markers in Klk1 AAV model combined with 2%DSS. All data are shown as the mean ± SEM. Data are representative of three independent experiments. The *P* value was analyzed by one‐way ANOVA with Tukey's multiple comparisons, and all *P* values are marked with specific values in the graph.

Next, we used the AAV2 (serotype 2) virus to knock down KLK1 in a 2% DSS‐induced colitis model to study its role under inflammatory conditions. We first performed an AAV enema 3 weeks in advance, that is, to ensure that the AAV virus knocked down the endogenous KLK1 successfully, and then drank 2% DSS (**Figure** **3**A). Colonoscopy of mice showed that drinking 2% DSS aggravated bleeding in the case of KLK1 knockdown (Figure 3B), KLK1 knockdown exacerbated the shortening of colon length (Figure 3C,D), as well as colon inflammation, disappearance of glandular abscesses, increased inflammatory cell infiltration, and mucosal destruction (Figure 3E,F). The expression of KLK1 in serum was further reduced (Figure 3G). The above results all showed that in the case of KLK1 knockdown alone and without 2% DSS, mice also had a certain degree of intestinal damage, and on this basis, mice drinking 2% DSS had more severe intestinal damage than mice drinking only 2% DSS.

In order to confirm the knockdown efficiency of KLK1 in colon tissue, immunofluorescence showed that KLK1 expression was reduced under 2% induced colitis conditions. (Figure 3H,I). In addition, we performed Western blot (WB) analysis on the colon protein lysates of AAV2‐KLK1 mice. Compared with AAV negative control (AAV‐NC) mice, the KLK1 protein level in the colon tissue of the knockdown group was significantly reduced, confirming the effective tissue level reduction (Figure 3J). We analyzed the FITC‐dextran permeability of the AAV2‐Klk1 knockdown model to evaluate the permeability of the intestinal epithelial barrier and found that Klk1 knockdown significantly increased the permeability of the intestine (Figure 3K). We further observed the changes in intestinal mucosal barrier‐related markers. After knocking down KLK1 in the intestine using AAV2, immunohistochemistry results showed that the expression of intestinal mucosal barrier markers was further reduced (Figure 3L,M). We performed protein quantitative analysis on the above results and obtained the same results. The Klk1, Zo‐1, E‐cadherin, and Occludin protein levels in the colon tissue were significantly reduced, confirming that the loss of KLK1 caused the intestine to lose the protection of the mucosal barrier (Figure 3N–R).

In summary, by knocking down endogenous KLK1 with AAV and combining it with the 2% DSS‐induced acute colitis model, we found that as inflammation intensified, the expression of KLK1 in colon tissue, serum, and intestinal epithelium further decreased, the intestine lost the protective effect of KLK1, and intestinal barrier markers further decreased. Knockdown of KLK1 further aggravated DSS‐induced colon injury and mucosal barrier disruption. These data suggest that endogenous KLK1 may protect the intestine by maintaining intestinal mucosal barrier function.

### 2.4 KLK1 Supplementation Can Alleviate Mucosal Barrier Damage Caused by DSS‐Induced Colitis

At present, some drugs have been widely used to treat inflammatory bowel disease (IBD) and have achieved certain efficacy. However, for some patients, existing treatments may have limitations, such as poor tolerability, large side effects or poor efficacy. Therefore, it is crucial to find new drugs or treatments to meet the needs of patients.

**Figure 4:**
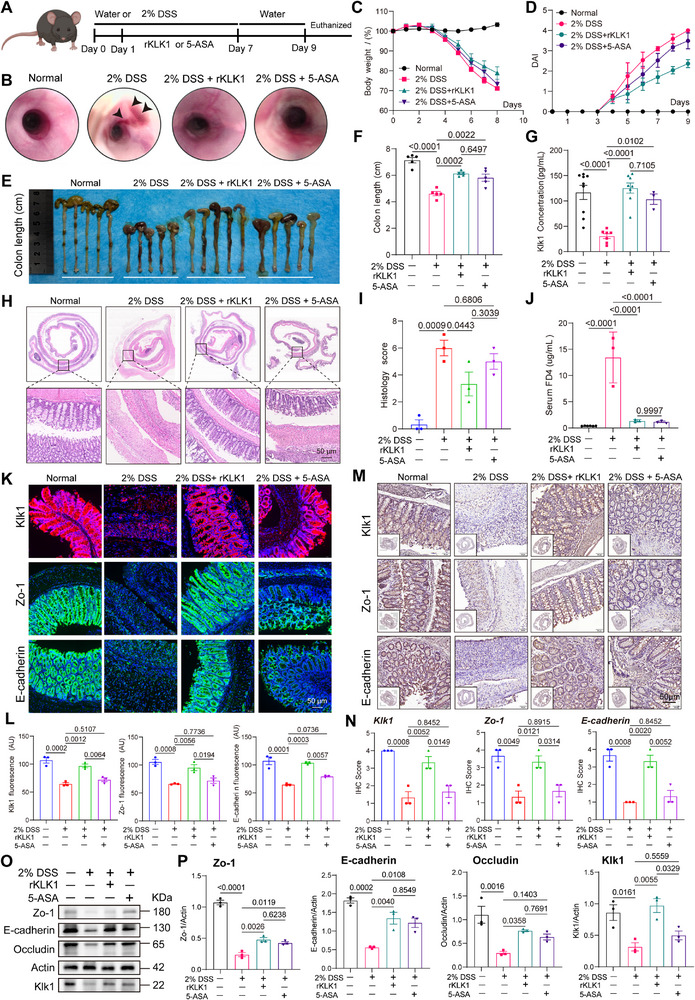
KLK1 can improve the mucosal barrier damage caused by DSS‐induced acute colitis. A) Schematic diagram of the treatment of DSS‐induced acute colitis with rKLK1 and 5‐ASA, respectively. B) High‐resolution endoscopic images of the colon in C57BL/6 mice with DSS‐induced acute colitis treated with rKLK1 and 5‐ASA, respectively. C) Body weight curves denote the changes in the mean body weight of mice recorded daily in different groups. D) Disease activity index (DAI) score of C57BL/6 mice colon with DSS‐induced acute colitis treated with rKLK1 and 5‐ASA.E) Representative picture of the colons from different groups (n=5 per group) and F) colon length. G) Detection of mouse serum Klk1 concentration by ELISA (n > 3 per group). H) Photomicrographs show representative images of H&E staining (n=3 per group). I) The histopathological grading of inflammation in C57BL/6 mice colon with DSS‐induced acute colitis treated with rKLK1 and 5‐ASA was recorded. J) FITC‐Dextran 4 (FD4) was used to detect intestinal permeability in C57BL/6 mice (n > 3 per group). K,L) Immunofluorescence staining of KLK1, Zo‐1, and E‐cadherin in C57BL/6 mice colon with KLK1 (red), Zo‐1 (green), E‐cadherin (green), and DAPI (blue) antibodies (n=3 per group). M,N) Immunohistochemistry of Swiss rolls showing the changes in the expression of KLK1 and intestinal epithelial mucosal barrier markers in the treatment of DSS‐induced acute colitis with rKLK1 and 5‐ASA, respectively (n=3 per group). O, P) Protein levels of Klk1, Zo‐1, E‐cadherin and Occludin in C57BL/6 mice colon with DSS‐induced acute colitis treated with rKLK1 and 5‐ASA, respectively. Scale bars: 50 µm. All data are shown as the mean ± SEM. Data are representative of three independent experiments. The *P* value was analyzed by one‐way ANOVA with Tukey's multiple comparisons, and all *P* values are marked with specific values in the graph.

We used the 2% DSS‐induced acute colitis model in mice, and used 5‐aminosalicylic acid (5‐ASA) as a positive control, which mainly exerts local anti‐inflammatory effects by acting on the intestinal mucosa, to explore the role of recombinant KLK1 (rKLK1) in colitis (**Figure** **4**A). Colonoscopy of mice showed that compared with the normal group, colonoscopy of mice in the model group showed obvious bleeding spots, while rKLK1 significantly improved colon involvement (Figure 4B). Compared with the normal group, the body weight of mice in the model group decreased by 10–30%, the colon length was shortened, and there were varying degrees of blood in the stool; on the basis of the model group, the colon length was restored, the body weight also increased to varying degrees, and the disease activity index (DAI) was reduced. rKLK1 can significantly improve the degree of colon inflammation in mice (Figure 4C–F). Compared with the normal group, the serum KLK1 level of mice in the model group was significantly reduced, which was partially restored after treatment with rKLK1 or 5‐ASA (Figure 4G). Acute colitis is manifested by colon mucosal destruction, irregular glandular arrangement, inflammatory cell infiltration, and ulcer formation, and the above changes were improved in the treatment group (Figure 4H,I). The serum FITC‐dextran level of mice in the model group increased and decreased after treatment, indicating that rKLK1 can improve colon permeability (Figure 4J). The expression of the KLK1 protein was low in the model group and significantly increased in the treatment group. The expression of Zo‐1, E‐cadherin, and Occludin was reduced in the DSS group and returned to normal after treatment (Figure 4K–N). In addition, we verified the above results by protein quantification (Figure 4O,P).

In the above results, we explored the therapeutic effect of rKLK1 in the DSS‐induced acute colitis model. The results showed that rKLK1 significantly improved colon inflammation, upregulated serum KLK1 levels, repaired intestinal barrier function, and reduced intestinal permeability. Histological analysis showed that rKLK1 reduced colon mucosal destruction, inflammatory cell infiltration, and ulcer formation. These results suggest that rKLK1 may play a therapeutic role by protecting the integrity of the intestinal barrier and reducing inflammatory responses, providing a new direction for potential treatment strategies for IBD.

### 2.5 KLK1‐B1R Axis Protects Intestinal Barrier Integrity and Suppresses Fibroblast‐Driven Inflammation‐Cancer Transition

**Figure 5:**
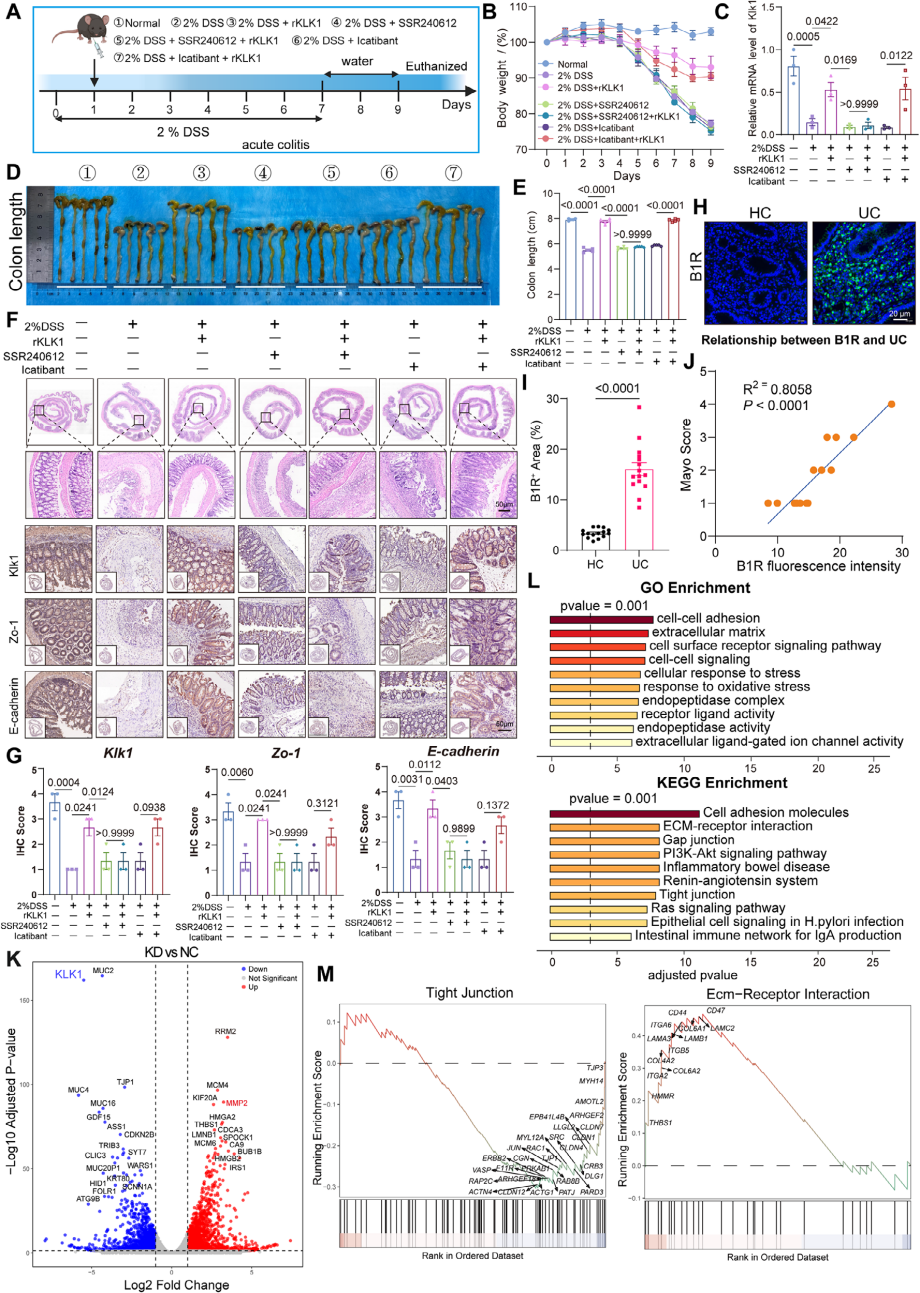
KLK1 protects the intestinal mucosal barrier through B1R. A) Schematic diagram of the treatment of DSS‐induced acute colitis with rKLK1 and different receptor inhibitors, including B1R inhibitor SSR240612 and B2R inhibitor Icatibant. B) Body weight curves denote the changes in the mean body weight of mice recorded daily in different groups. C) Relative mRNA level of Klk1 in different C57BL/6 mice colon groups. All data are expressed as mean ± SEM from three independent experiments (n = 3 per group). D) Representative picture of the colons from different groups and E) colon length (n=5 per group). F,G) Immunohistochemistry of Swiss rolls shows changes in the expression of Klk1 and intestinal epithelial barrier markers in B1R and B2R inhibitor models combined with 2% DSS‐induced acute colitis and rKLK1. Scale bars: 50 µm (n = 3 per group). H,I) Immunofluorescence staining of BDKRB1^+^ cells in healthy controls (HC) and ulcerative colitis (UC) patients, BDKRB1 (green) and DAPI (grey) antibodies. Patient information is provided in Table S4 (Supporting Information). Scale bars: 20 µm. (n = 15 per group). J) The immunofluorescence intensity of BDKRB1 is significantly positively correlated with the Mayo score of ulcerative colitis. K) Volcano plot showing differentially expressed genes in KLK1 after NCM460 knockdown (KD) relative to the negative control (NC). L) Bar plot showing GO and KEGG representative pathways enriched by RNA‐Seq after KLK1 knockdown in NCM460 cell lines. M) GSEA enrichment analysis showed that KLK1 significantly downregulated the tight junction pathway and upregulated the ECM pathway after NCM460 knockdown (KD) compared with the negative control (NC). All data are shown as the mean ± SEM. Data are representative of three independent experiments. The *P* value was analyzed by one‐way ANOVA with Tukey's multiple comparisons, and all *P* values are marked with specific values in the graph.

B1R and B2R bind to kinin‐derived peptide hormones and mediate transmembrane (TM) signaling primarily through the Gq pathway. However, kinins differ in their selectivity for bradykinin receptor subtypes, so we wanted to explore whether KLK1 metabolites play a protective role in the intestinal mucosal barrier primarily through B1R or B2R. Next, based on an acute enteritis animal model, we established a B1R inhibitor group, SSR240612, and a B2R inhibitor group, Icatibant (**Figure** **5**A). Regardless of whether B2R was inhibited, colon length and weight were restored after KLK1 administration (Groups 6 and 7), but not after B1R inhibition (Groups 4 and 5). Compared with the model group, only groups 2 and 7 showed significantly increased KLK1 mRNA levels, indicating that KLK1 metabolites act through B1R rather than B2R (Figure 5B–E). In the 2% DSS group, KLK1, Zo‐1, and E‐cadherin expression were reduced. Supplementation with rKLK1 restored these markers, but inhibition of B1R (SSR240612) further impaired the intestinal barrier, which rKLK1 could not rescue. Inhibition of B2R reduced intestinal barrier‐related markers, but rKLK1 restored these markers, confirming that KLK1 exerts a protective effect through B1R rather than B2R (Figure 5F,G). Next, we collected pathological sections from 15 healthy controls (HC) and 15 patients with ulcerative colitis (UC). Immunofluorescence results showed a significant increase in B1R^+^ cells in UC tissues, and quantitative analysis confirmed its statistical significance (Figure 5H,I). We also performed a correlation analysis based on the immunohistochemical score of BDKRB1 in ulcerative colitis and the Mayo score of the corresponding patients. The results showed that the IHC score of BDKRB1 was significantly positively correlated with the Mayo score of ulcerative colitis (Figure 5J). These results illustrate the key role of the KLK1‐B1R axis in maintaining intestinal barrier homeostasis and provide new directions for targeted therapy.

The above experimental results gave us new ideas. Which signaling pathways would be activated by the reduction of KLK1? Next, we knocked down KLK1 in the normal human colon cell line NCM460 and analyzed it by RNA‐Seq. The volcano plot results showed that after KLK1 knockdown, TJP1, MUC2, MUC4, and MUC16 (Mucin family members, mucin) were all downregulated, and genes related to proliferation and migration (RRM2, THBS1, LMNB1) and genes related to matrix reprogramming (MMP2, MMP9) were all upregulated (Figure 5K). Consistent with this, GO and KEGG showed that the main enriched pathways after KLK1 downregulation were cell adhesion, tight junctions, oxidative stress, and extracellular matrix (Figure 5L). GSEA analysis showed that after knocking down KLK1, the tight junction pathway was significantly downregulated, further proving that KLK1 knockdown may lead to weakened epithelial barrier function, adhesion/tight junction destruction, and weakened mucus protection function, suggesting that it is involved in regulating epithelial homeostasis and mucosal barrier integrity, while the extracellular matrix (ECM) pathway was significantly upregulated (Figure 5M). In subsequent studies, we will focus on the ECM pathway.

### 2.6 KLK1 Depletion Promotes Colorectal Inflammation‐Cancer Transformation by Activating ECM Pathways via B1R

**Figure 6:**
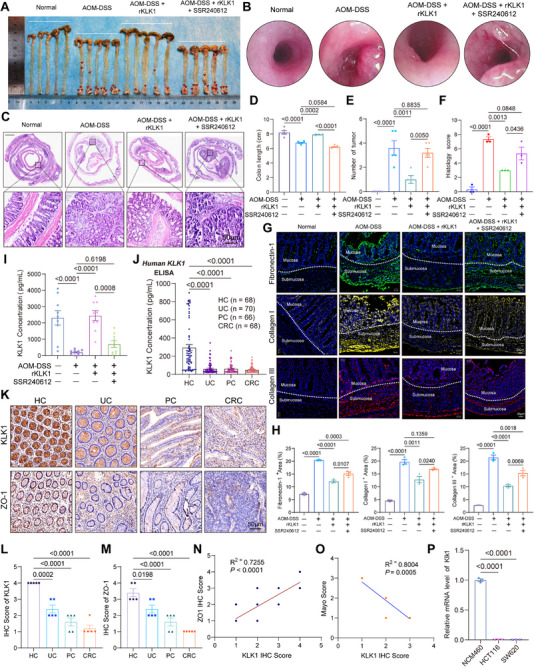
KLK1 downregulation promotes fibroblast phenotype transformation. A) Representative picture of the C57BL/6 mice colons from the AOM‐DSS‐induced inflammation‐cancer transformation model and treatment with rKLK1 or rKLK1 combined with SSR240612, respectively (n= 5 per group). B) High‐resolution endoscopic images of the colon in C57BL/6 mice with AOM‐DSS‐induced inflammation‐cancer transformation model and treatment with rKLK1 or rKLK1 combined with SSR240612, respectively. C) Photomicrographs show representative images of H&E staining (n=3 per group). D–F) The colon length (D), number of tumors (E), and histology score (F) in C57BL/6 mice with AOM‐DSS‐induced inflammation‐cancer transformation model and treatment with rKLK1 or rKLK1 combined with SSR240612, respectively. G,H) Immunofluorescence staining of Fibronectin (green), Collagen I (yellow), Collagen III (red), and DAPI (blue) antibodies in AOM‐DSS‐induced inflammation‐cancer transformation model and treatment with rKLK1 or rKLK1 combined with SSR240612, respectively. Scale bars: 20 µm. (n=3 per group). I) Detection of mouse serum Klk1 concentration by ELISA (n=9 per group). J) Detection of human serum KLK1 concentration by ELISA during different group of the development of colorectal cancer. Patient information is provided in Table S3 (Supporting Information). K–M) Representative images of IHC staining for KLK1 and ZO‐1 during different group of the development of colorectal cancer and L,M) Data are representative of five independent experiments. Patient information is provided in Table S4 (Supporting Information) (n=5 per group). N) Pearson correlation analysis of IHC scores of KLK1 and ZO‐1. O) Pearson correlation analysis between the IHC score of KLK1 and the corresponding Mayo score of patients with ulcerative colitis. P) Relative mRNA level of KLK1 in different cell lines. All data are shown as the mean ± SEM. Data are representative of three independent experiments. The *P* value was analyzed by one‐way ANOVA with Tukey's multiple comparisons, and all *P* values are marked with specific values in the graph.

In the colitis‐carcinoma transformation model, KLK1 supplementation reduced adenoma formation induced by AOM‐DSS, an effect inhibited by SSR240612. Endoscopy showed KLK1 treatment significantly improved adenoma occurrence, indicating KLK1 protects the gut and prevents inflammation‐cancer transformation via B1R (**Figure** **6**A,B). Colon histology revealed mucosal damage, irregular gland arrangement, inflammatory infiltration, and carcinogenesis, which were alleviated by KLK1 but worsened with B1R inhibition (Figure 6C–F). The intestinal mucosal barrier is closely related to the ECM, and their interaction is essential for the structure, function, and health of the gut.^[^ ^12^ ^]^ Fibronectin‐1, type I collagen, and type III collagen were significantly upregulated in the mucosa and submucosa of AOM‐DSS mice. KLK1 administration significantly reversed the accumulation of ECM and enhanced its anti‐fibrotic and matrix remodeling effects in the context of tumor growth promotion, while administration of the B1R inhibitor SSR240612 on this basis found that ECM‐related indicators were not significantly restored, indicating that the conduction of the KLK1‐B1R axis signaling axis was hindered. These data support our hypothesis that KLK1 regulates ECM remodeling in the acute and chronic colitis environment and provide functional evidence for the regulatory role of KLK1 in fibrosis‐related matrix remodeling (Figure 6G,H).

Chronic inflammatory diseases are often associated with an increased risk of cancer.^[^ ^2^ ^]^ Serum KLK1 changes in mice aligned with these findings (Figure 6I). In addition, we collected serum from 272 clinical patients at four stages, including healthy controls (HC), ulcerative colitis (UC), pre‐cancer (PC), and colorectal cancer (CRC) patients, and detected the expression of KLK1 in serum. We found that with the progression of disease stage, KLK1 expression was higher in healthy patients, while with the progression of disease stage, KLK1 expression gradually decreased (Figure 6J). We collected 4 stages of colorectal cancer progression from the clinic, 5 patients in each stage, a total of 20 patients, and analyzed the KLK1 expression level with the intestinal barrier integrity marker ZO‐1 and the validation score of ulcerative colitis patients: Immunohistochemistry (IHC) of pathological sections confirmed that KLK1 and ZO‐1 protein levels were expressed most highly in healthy controls, and KLK1 expression decreased significantly with inflammatory cancer transformation (Figure 6K–M). In addition, we observed a significant positive correlation between KLK1 and ZO‐1 IHC scores (R^2^ = 0.7255, P < 0.0001, Figure 6N), indicating that KLK1 expression is closely related to epithelial barrier integrity. We also performed a correlation analysis based on the immunohistochemical score of KLK1 in ulcerative colitis and the Mayo score of the corresponding patients, and found a significant negative correlation between the two (R^2^ = 0.8004, P = 0.0005), indicating that lower KLK1 levels are associated with more severe inflammation (Figure 6O). All of the above results further support the protective role of KLK1 on the intestinal mucosal barrier. KLK1 was highly expressed in normal colon cells (NCM460) but low or absent in colorectal cancer cells (HCT116, SW620) (Figure 6P).

In summary, we used the AOM‐DSS‐induced colitis‐cancer transformation model to reveal the mechanism of action of KLK1 in inhibiting adenoma formation and maintaining intestinal homeostasis through a B1R‐dependent mechanism. These results clarify the molecular mechanism by which the KLK1‐B1R axis inhibits inflammation‐cancer transformation by regulating fibroblast phenotypic transformation and ECM dynamic balance, providing a new strategy for targeted intervention. These results link KLK1 to intestinal mucosal barrier function and disease severity in human IBD, thereby enhancing the translational relevance of our findings.

### 2.7 Single‐Cell RNA Sequencing of AOM‐DSS‐Induced Colitis‐Cancer Transformation

**Figure 7:**
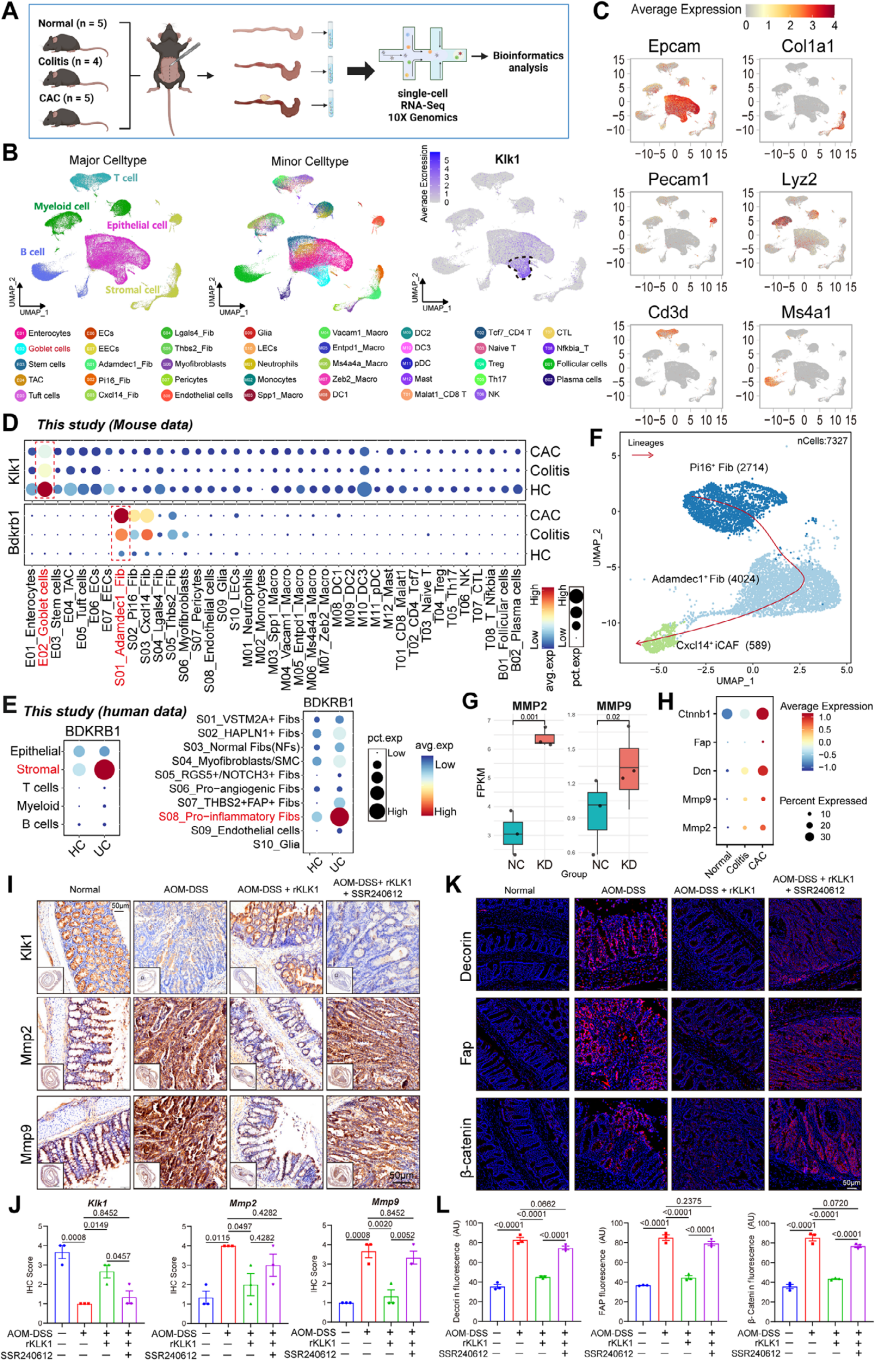
Analysis of KLK1 expression in AOM‐DSS‐induced inflammation‐cancer transformation scRNA‐seq dataset. A) The schematics of sample collection, scRNA‐seq transcriptomic analysis of the AOM‐DSS‐induced inflammation‐cancer transformation model. B) UMAP of 93 158 cells analyzed by scRNA‐seq across 14 samples. Clusters were annotated by the canonical markers and a feature plot showing the expression of Klk1 in all cells. C) Feature plot showing the expression levels of the selected markers in all cell subtypes. D) The expression levels of Bdkrb1 and Klk1 in different cell subtypes during the scRNA‐seq dataset of C57BL/6 mice transformed by AOM‐DSS‐induced inflammation and cancer. Dot size indicates the fraction of expressing cells, and the colors represent normalized gene expression levels. E) The expression levels of B1R in different cell subtypes and fibroblast subtypes. Dot size indicates the fraction of expressing cells, and the colors represent normalized gene expression levels. F) AOM‐DSS‐induced inflammation‐cancer transformation model scRNA‐seq data analysis of pseudotime differentiation trajectories of fibroblast subsets in the in C57BL/6 mice. G) RNA‐seq data showed FPKM expression of MMP2 and MMP9 in KLK1‐knockdown (KD) relative to negative control (NC). H) Dot plot showing the expression levels of Ctnnb1, Fap, Dcn, Mmp2, and Mmp9 in different groups of AOM‐DSS‐induced inflammation‐cancer transformation model scRNA‐seq data. Dot size indicates the fraction of expressing cells, and the colors represent normalized gene expression levels. I,J) Representative images of IHC staining for Klk1, Mmp2, and Mmp9 during different groups (n=3 per group). K,L) Immunofluorescence staining of β‐catenin (red), Fap (red), Decorin (red), and DAPI (blue) antibodies in AOM‐DSS‐induced inflammation‐cancer transformation model and treatment with rKLK1 and SSR240612, respectively (n=3 per group). Scale bars: 50 µm. All data are shown as the mean ± SEM. The *P* value was analyzed by one‐way ANOVA with Tukey's multiple comparisons, and all *P* values are marked with specific values in the graph.

To explore the expression of KLK1 and B1R in the colitis‐cancer transformation model, we constructed an AOM‐DSS‐induced colitis‐cancer transformation model in mice, obtained intestinal tissues for single‐cell sequencing (**Figure** **7**A), identified cell subpopulations and performed dimensionality reduction clustering analysis, and divided the cells into five subpopulations: epithelial cells, stromal cells, T cells, B cells, and myeloid cells and found that Klk1 was highly expressed specifically in goblet cells (Figure 7B,C). In addition, we found that in the single‐cell dataset of the AOM‐DSS‐induced inflammatory cancer transformation model, the expression of Bdkrb1 gradually increased with the reduction of KLK1, especially in the Adamdec1^+^ fibroblast subpopulation (Figure 7D). In the ulcerative colitis single‐cell dataset, we found that BDKRB1 expression was significantly increased in UC and was mainly expressed in the pro‐inflammatory fibroblast subpopulation (Figure 7E). Therefore, we verified the co‐localization of BDKRB1 and ADAMDEC1 in pathological sections of patients with colorectal adenoma (Figure S2A–C, Supporting Information). These findings suggest that the loss of KLK1 expression may promote inflammation‐related cancer transformation by activating the Bdkrb1⁺Adamdec1⁺ fibroblast subpopulation, which provides a new perspective on the potential mechanism of intestinal inflammation progression to cancer.

Next, we performed pseudotime analysis of the B1R‐expressing fibroblast subgroup, and the Pi16⁺ fibroblast subset served as the initiation end of differentiation, while the Adamdec1⁺ fibroblast subset differentiated as an intermediate transition state to the Cxcl14⁺ inflammatory cancer‐associated fibroblasts (iCAFs), indicating that the Bdkrb1⁺Adamdec1⁺ fibroblast subset underwent an inflammatory‐carcinoma phenotypic transition (Figure 7F). There is a report showing that decorin, a component of the extracellular matrix, differed most significantly among the four inflammatory CAF subtypes, and patients with high decorin expression on pre‐treatment biopsies had significantly worse DFS compared to those with low expression.^[^ ^13^ ^]^ Specific Fap⁺ CAFs subsets have been identified in solid tumors in multiple laboratories worldwide. Importantly, this Fap⁺ CAFs subset accumulates in cancers with poor prognosis and has been shown to be associated with metastatic spread and immunosuppression of cancer.^[^ ^14^, ^15^, ^16^ ^]^

Previous studies have shown that KLK1 knockdown may activate the ECM pathway through upregulation of B1R, and RNA‐seq data showed that genes related to matrix reprogramming (MMP2, MMP9) were upregulated in the KLK1‐knockdown (KD) group (Figure 7G). Through single‐cell data analysis, the expression of markers associated with the ECM pathway and fibroblast phenotypic transition increased gradually during inflammation‐cancer transformation (Figure 7H). Expression of Mmp2 and Mmp9 is associated with immune markers, and they play an important role in epithelial‐mesenchymal transition (EMT) and immune response.^[^ ^17^ ^]^ In the colitis‐cancer transformation model, KLK1 expression was significantly reduced. Increased KLK1 expression led to decreased Mmp2 and Mmp9 levels and reduced adenoma formation, an effect inhibited by SSR240612 (Figure 7I,J). In the previous results, we found that the expression of E‐cadherin, the main molecule of cell‐cell adhesion, is downregulated in the colitis model, and it links cells together by β‐catenin, thereby maintaining the structure and function of epithelial cells. In CRC, E‐cadherin is often downregulated or lost, resulting in altered interactions between β‐catenin and ECM, which in turn promote the detachment and invasion of tumor cells.^[^ ^18^ ^]^ In addition, β‐catenin can also regulate the synthesis and remodeling of the ECM. Studies have shown that β‐catenin affects the composition and structure of the ECM by regulating the expression of some genes in tumor cells. For example, β‐catenin can promote the degradation of the ECM by activating MMPs (matrix metalloproteinases) or collagenases, providing conditions for the migration and invasion of tumor cells.^[^ ^19^, ^20^ ^]^ Decorin, Fap, and β‐catenin were significantly upregulated in the inflammation‐cancer transformation model group, while KLK1 treatment significantly reduced their expression, and this improvement was inhibited in the SSR240612 group (Figure 7K,L).

In summary, our study revealed that KLK1 plays an important protective role in preventing inflammation‐driven colorectal cancer progression. Using single‐cell RNA sequencing of the AOM‐DSS‐induced colitis‐cancer model, we found that KLK1 was specifically expressed in goblet cells, and its reduced expression was accompanied by increased BDKRB1 expression in ADAMDEC1⁺ fibroblasts, an inflammatory subset associated with cancer transformation. Pseudo‐temporal analysis further demonstrated that Bdkrb1⁺Adamdec1⁺ fibroblasts represent a transitional population evolving toward iCAFs. Mechanistically, loss of Klk1 promoted extracellular matrix (ECM) remodeling by upregulating Mmp2, Mmp9, Fap, decorin, and β‐catenin, thereby promoting epithelial‐mesenchymal transition and tumor progression. Notably, Klk1 treatment suppressed these pro‐tumorigenic changes, while the B1R antagonist SSR240612 reversed this effect, highlighting the KLK1‐B1R axis as a potential therapeutic target for inflammation‐associated colorectal cancer.

### 2.8 KLK1‐B1R Axis Inhibits Colorectal Adenoma Carcinogenesis by Regulating the Wnt/β‐Catenin Pathway

In order to comprehensively evaluate the role of KLK1 in the development and progression of colorectal cancer, we used two classic mouse models. On the one hand, the AOM‐DSS model simulates the process of colorectal cancer transformation driven by chronic inflammation, which helps to explore the function of KLK1 in inflammation‐related tumorigenesis. On the other hand, the *Apc* ^min/+^ mouse model represents the process of adenoma carcinogenesis driven by gene mutations, which can be used to verify the prevalence and mechanism of KLK1 in non‐inflammation‐dependent colorectal tumorigenesis. Through these two complementary models, we can more systematically analyze the role of KLK1 in different carcinogenic pathways, thereby revealing its value as a potential therapeutic target. The *Apc* ^min/+^ model drives adenoma‐carcinogenesis through APC gene mutation‐induced hyperactivation of the Wnt/β‐catenin signaling pathway.

**Figure 8:**
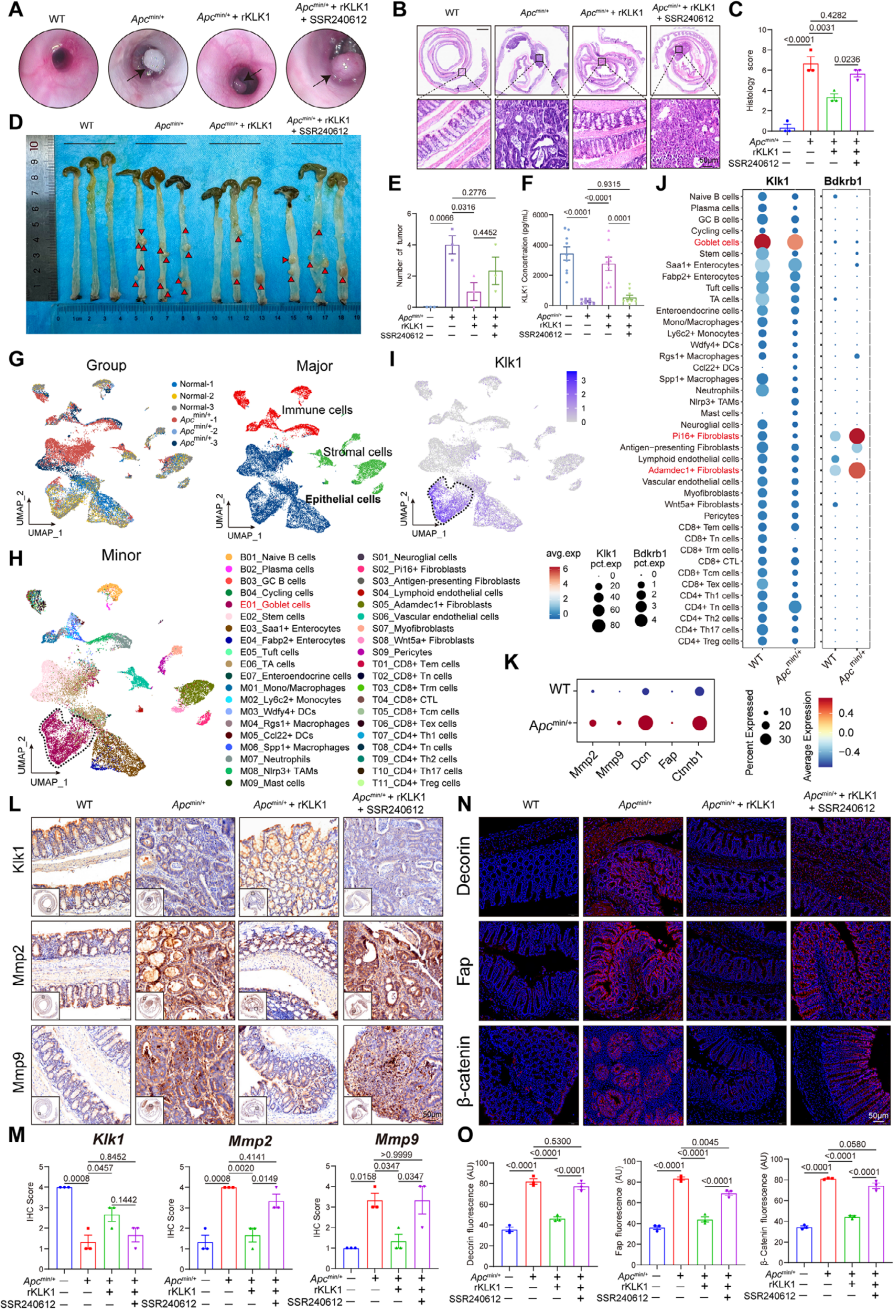
KLK1 supplementation prevents adenomatous carcinogenesis in APC‐deficient intestine. A) High‐resolution endoscopic images of the colon in *Apc* ^min/+^ mice which treatment with rKLK1 or rKLK1 combined with SSR240612, respectively. B) Photomicrographs show representative images of H&E staining and C) The histology score (n = 3 per group). D) Representative picture of the *Apc* ^min/+^ mice colons in treatment with rKLK1 or rKLK1 combined with SSR240612, respectively, and E) Number of tumors (n = 3 per group). F) Detection of mouse serum Klk1 concentration by ELISA (n > 3 per group). G) UMAP of wild type or *Apc* ^min/+^ mice analyzed by scRNA‐seq across 6 samples, and H) Clusters were annotated by the canonical markers. I) UMAP plot showing the expression levels of Klk1 in all cell subtypes. J) The expression levels of Klk1 and Bdkrb1 in different cell subtypes, and K) Ctnnb1, Fap, Dcn, Mmp2, and Mmp9 in different groups. Dot size indicates the fraction of expressing cells, and the colors represent normalized gene expression levels. L,M) Representative images of IHC staining for Klk1, Mmp2, and Mmp9 during different groups (n = 3 per group). N,O) Immunofluorescence staining of β‐catenin (red), Fap(red), Decorin (red), and DAPI (blue) antibodies in *Apc* ^min/+^ mice, which treatment with rKLK1 and SSR240612, respectively (n = 3 per group). Scale bars: 50 µm. All data are shown as the mean ± SEM. Data are representative of three independent experiments. The *P* value was analyzed by one‐way ANOVA with Tukey's multiple comparisons, and all *P* values are marked with specific values in the graph.

Next, we collected samples from the high‐fat diet (HFD)‐induced *Apc* ^min/+^ adenoma carcinogenesis model and performed scRNA‐seq analysis (Figure S3A, Supporting Information), and we found that in the *Apc* ^min/+^ mouse model, supplementation with KLK1 inhibited colon adenoma formation, while the B1R inhibitor SSR240612 reversed this protective effect (**Figure** **8**A). Histologically, the model group exhibited epithelial hyperplasia, disorganized glandular structures, and a marked increase in adenoma count within the colon. KLK1 administration significantly reduced adenoma incidence, but B1R inhibition attenuated this improvement (Figure 8B–E). ELISA analysis confirmed that serum KLK1 levels correlated with phenotypic outcomes (Figure 8F). Furthermore, cell subset identification and dimensionality reduction cluster analysis were used to divide the cells into three major subsets: epithelial cells, stromal cells, and immune cells (Figure 8G). Consistent with the results of previous studies, KLK1 was enriched specifically in goblet cells (Figure 8H,I), and goblet cell‐specific expression of KLK1 was decreased in the model group, while BDKRB1 showed high expression in Pi16^+^ and Adamdec1^+^ fibroblasts (Figure 8J). In the *Apc* ^min/+^ model, adenoma carcinogenesis is usually caused by mutations in the APC gene, which leads to aberrant activation of the Wnt/β‐catenin signaling pathway, thereby promoting intestinal epithelial cell proliferation, abnormal differentiation, and tumorigenesis.^[^ ^21^ ^]^ Mmp2, Mmp9, Dcn, Fap, and Ctnnb1 were up‐regulated in the *Apc* ^min/+^ model, and KLK1 was significantly down‐regulated (Figure 8K), we verified the expression of these genes and found that the expression of Mmp2 and Mmp9 was significantly reduced in the KLK1‐supplemented group, and the administration of the B1R inhibitor SSR240612 reversed this protective effect (Figure 8L–O).

In summary, we demonstrated that KLK1‐B1R plays a broad inhibitory role in colorectal tumor formation using two classic colorectal cancer models, the AOM‐DSS inflammation‐oncogenic transformation model and the *Apc* ^min/+^ adenoma carcinogenesis model. In the *Apc* ^min/+^ model, supplementation with KLK1 significantly inhibited adenoma formation, reduced epithelial dysplasia, and downregulated pro‐oncogenes such as Mmp2, Mmp9, Fap, Dcn, and Ctnnb1. Single‐cell analysis further revealed that KLK1 remained enriched in goblet cells, while Bdkrb1 was upregulated in Pi16⁺ and Adamdec1⁺ fibroblasts, consistent with the findings in the inflammation model. Notably, the protective effect of KLK1 was reversed by B1R inhibition, highlighting the conservation of the KLK1‐B1R regulatory axis in different oncogenic pathways. These results highlight the therapeutic potential of KLK1 in both inflammation‐dependent and ‐independent colorectal cancers.

In order to further compare the similarities and differences of cellular communication networks in the tumor microenvironment under different carcinogenic mechanisms, we performed CellChat^[^ ^22^ ^]^ analysis on the AOM‐DSS inflammation‐related carcinogenesis model and the *Apc* ^min/+^ gene‐driven adenoma model based on single‐cell transcriptome data. We compared the total number and intensity of interactions in the *Apc* ^min/+^ adenoma carcinogenesis model through interaction analysis and found that both were reduced (Figure S3B, Supporting Information). The total number of cell communication pairs in wild‐type mice (WT) was 234, while that in the *Apc* ^min/+^ model was 216; the communication intensity in WT was 0.352, while that in the *Apc* ^min/+^ group was significantly reduced to 0.173; indicating that in the *Apc* ^min/+^ model, the overall cell‐to‐cell communication was weakened. Next, we used the chord diagram to show the number of communications between the five major blast subsets and goblet cells in the normal group and the CAC model. In CAC, the interaction between goblet cells and Adamdec1⁺ Fibroblasts was significantly weakened. The same results were obtained in the *Apc* ^min/+^ model. The interaction between Adamdec1⁺ fibroblasts and Goblet cells was significantly weakened in the model (Figure S3C,D, Supporting Information). Goblet cells are responsible for secreting mucus, forming an intestinal barrier that protects intestinal epithelial cells from harmful substances.^[^ ^23^ ^]^ Adamdec1^+^ fibroblasts help support goblet cell function and intestinal barrier stability.^[^ ^24^ ^]^ We compared the key information flows of CAC versus Normal and found that MMP was upregulated in CAC while KLK‐related pathways were significantly weakened in CAC (Figure S3E, Supporting Information). Next, we showed the signal flow of KLK and MMP signaling pathways between fibroblast subsets and Goblet cells, and found that KLK signals were mainly sent from Goblet cells to Adamdec1⁺ fibroblasts; while MMP was mainly active among fibroblast subsets, indicating its important role in ECM remodeling (Figure S3F,G, Supporting Information). We further analyzed the role distribution (Sender/Receiver/Mediator/Influencer) in the KLK and MMP signaling pathways and found that Goblet cells are the main senders of KLK, Adamdec1⁺ fibroblasts are the main receivers and mediators. In different fibroblast subsets, the roles of KLK and MMP signals were once again clarified, highlighting that KLK is an important factor in regulating the ECM microenvironment (Figure S3H,I, Supporting Information).

In summary, we demonstrated that the KLK pathway is active in the signaling axis between goblet cells and fibroblasts, while the MMP pathway dominates the ECM remodeling communication between fibroblasts. In tumor progression, the communication pattern of KLK downregulation and MMP upregulation may be the core mechanism by which inflammation and mutation jointly drive tumor microenvironment remodeling. Cell communication analysis further emphasized the role of Adamdec1⁺ fibroblasts as key receivers and mediators of proinflammatory and matrix regulatory signals. The above results emphasize the functional importance of the KLK1‐BDKRB1 axis and fibroblast reprogramming in shaping the pro‐tumor microenvironment during inflammation‐ and mutation‐driven colorectal carcinogenesis.

### 2.9 EGR1 Identified as Key Transcriptional Regulator of KLK1 in Colitis‐Associated Cancer Pathogenesis

**Figure 9:**
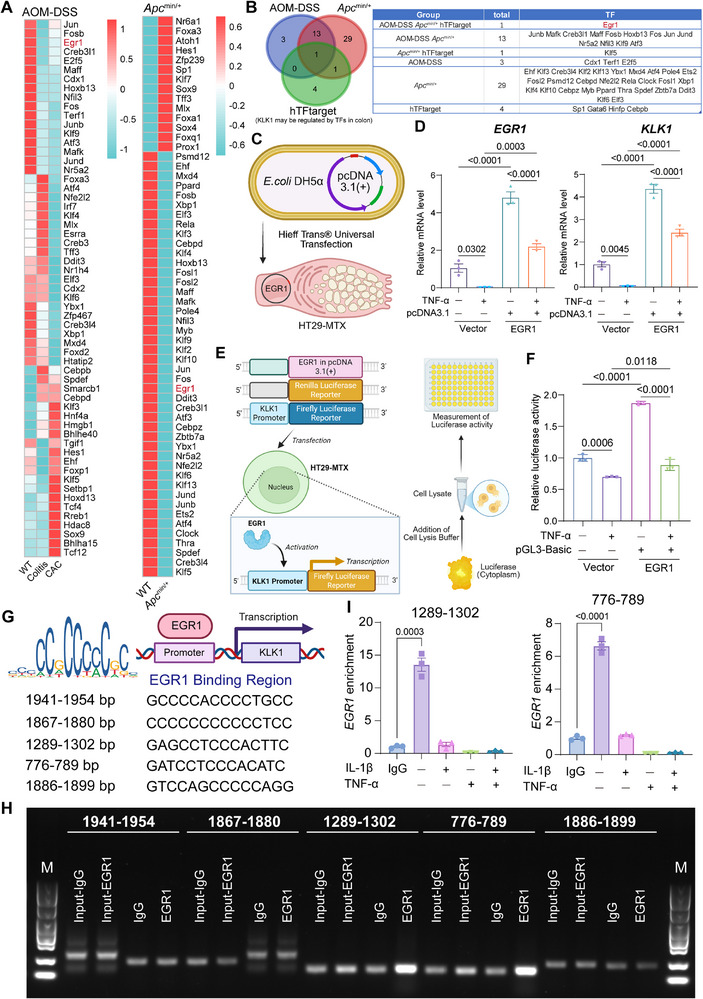
The decrease of KLK1 may be due to the transcriptional regulation by EGR1. A) Heatmaps showing transcription factors enriched in epithelial cells of different groups using SCENIC analysis of the AOM‐DSS‐induced inflammation‐cancer transformation scRNA‐seq dataset (left) and the *Apc* ^min/+^ adenoma carcinoma scRNA‐seq dataset (right). B) The Venn diagram shows the intersection of transcription factors enriched in epithelial cells of different groups by SCENIC analysis of the AOM‐DSS‐induced inflammation‐cancer transformation scRNA‐seq dataset (left) and the *Apc* ^min/+^ adenoma carcinoma scRNA‐seq dataset (right), and the transcription factors predicted in the hTFtarget database that may regulate KLK1 in colon (bottom). The table shows the transcription factors in the Venn diagram. C,D) Schematic diagram of constructing EGR1 overexpression plasmid and transfecting it into goblet cell line HT29‐MTX (C) and qPCR verification of the relative mRNA expression levels of EGR1 and KLK1 under EGR1 overexpression and inflammatory factor stimulation (D). E,F) Schematic diagram of the dual luciferase reporter gene experiment of EGR1 and KLK1 (E) and the relative luciferase activity of KLK1 promoter binding under the conditions of EGR1 overexpression and inflammatory factor stimulation (F). G) The Jaspar database was used to predict the possible binding sites between EGR1 and the pre‐transcriptional start region of KLK1. H) The ChIP method was used to verify the binding site of EGR1 and KLK1. The results were expressed by PCR. I) qPCR showing EGR1 was significantly enriched under the stimulation of inflammatory factors. All data are shown as the mean ± SEM. Data are representative of three independent experiments. The *P* value was analyzed by one‐way ANOVA with Tukey's multiple comparisons, and all *P* values are marked with specific values in the graph.

Previous studies have found that KLK1 expression is significantly reduced during the process of inflammatory transformation, but its regulatory mechanism is not yet clear. In order to reveal the molecular basis of KLK1 expression, we will further explore the mechanism affecting KLK1 regulation from the transcriptional regulation level. We performed SCENIC^[^ ^25^, ^26^ ^]^ analysis through inflammation‐cancer transformation and *Apc* ^min/+^ adenoma carcinogenesis models, and hoped that a transcription factor that regulates KLK1 expression will be found (**Figure** **9**A). EGR1 was discovered by intersecting with transcription factors predicted by the hTFtarget database that may regulate KLK1 in the colon (Figure 9B).

To further confirm the regulatory effect of EGR1 on KLK1, we designed EGR1 overexpression/rescue experiments and luciferase reporter assays to further verify the regulatory relationship between EGR1 and KLK1. We constructed the EGR1 expression vector (pcDNA3.1‐EGR1) in E. coli DH5α. Because previous studies have shown that KLK1 is highly expressed in goblet cells, we extracted the EGR1 plasmid and transfected it into the human intestinal goblet cell line HT29‐MTX using Hieff Trans Universal Transfection reagent (Figure 9C). The results showed that, left (EGR1): vector group, TNF‐α stimulation can downregulate EGR1 expression, and when EGR1 is overexpressed, its mRNA level is further enhanced. Right (KLK1): vector group, KLK1 expression is significantly downregulated under TNF‐α stimulation. When EGR1 is overexpressed, the transcription level of KLK1 is significantly upregulated (P < 0.0001). The results in this part indicate that EGR1 can promote the expression of KLK1, suggesting that EGR1 may act as a transcriptional activator of KLK1, supporting the regulatory relationship between the two (Figure 9D).

In addition, we also constructed a luciferase reporter plasmid (Firefly luciferase) containing the KLK1 promoter, and co‐transfected the EGR1 expression plasmid with the renilla internal reference (Figure 9E). The schematic diagram shows the process of EGR1 binding to the KLK1 promoter region in the cell nucleus and activating the downstream reporter gene. After 48 h of co‐transfection, luciferase activity was detected to reflect promoter activity. The results showed that in HT29‐MTX cells, the EGR1 overexpression group significantly increased the luciferase activity of the KLK1 promoter (P < 0.0001), while the basic vector (pGL3‐Basic) had no significant activation effect. The basic vector group showed that the luciferase activity decreased under inflammatory conditions, while the EGR1 overexpression group suggested that it had a significant transcriptional activation effect on the KLK1 promoter (P = 0.0118). This experiment directly demonstrated that EGR1 can activate the expression of KLK1 through the promoter at the functional level (Figure 9F).

Subsequently, Jaspar Base was used to predict possible binding sites for EGR1 to the pre‐transcriptional initiation region of KLK1, which revealed five possible binding sites (Figure 9G). Furthermore, the method of ChIP‐PCR was used to design primers for specific sites for PCR to verify the binding sites of transcription factors. PCR results show that EGR1 may bind to positions 1289–1302 or 776–789 (Figure 9H). Expression of EGR1 is associated with tumor suppression due to cell cycle arrest and apoptosis by modulating tumor suppressor pathways, including PTEN. Patients with high EGR1 levels had better overall and disease‐free survival than those with low EGR1 levels, and low EGR1 expression predicted low survival.^[^ ^27^ ^]^ We examined EGR1 binding under cytokine stimulation, finding IL‐1β/TNF‐α weakened its binding to KLK1's promoter region, explaining KLK1's expression decline post‐inflammation. Therefore, EGR1 may act as a transcriptional promoter of KLK1 (Figure 9I).

These findings revealed for the first time that EGR1 is a key transcription factor regulating KLK1 expression, providing a molecular mechanism at the transcriptional level for KLK1 dysregulation, suggesting that the EGR1‐KLK1 axis may inhibit inflammatory‐cancer transformation by regulating pathways such as PTEN, providing a new strategy for targeted intervention.

### 2.10 The Supplementation of Lys‐Des‐Arg^9^‐BK Can Alleviate the Damage Caused by the Negative Feedback Upregulation of B1R

B1R is thought to be an important mediator of oxidative stress and inflammation. The upregulation of kinin B1R in inflammation models relies in part on TNF‐α, a well‐established target for IBD drug therapy.^[^ ^28^ ^]^ The therapeutic effect of anti‐TNF‐α agents such as infliximab in IBD may be due in part to the inhibition of kinin B1R expression, and selective kinin B1R antagonists have demonstrated potent anti‐inflammatory effects in IBD models.^[^ ^29^ ^]^ We analyzed scRNA‐seq data from the CCD‐18Co colon fibroblast cell line treated with various inflammatory factors,^[^ ^30^ ^]^ BDKRB1 expression was highest in IL‐1β and TNF‐α groups (Figure S4A–D, Supporting Information). Single‐cell analysis (≈7000 cells) revealed stronger BDKRB1 responsiveness and heightened inflammation under TNF‐α versus IL‐1β (Figure S4E–G, Supporting Information). Pathway enrichment (GSVA/GO) highlighted TNF‐α‐driven immune cell chemotaxis, whereas IL‐1β promoted leukocyte activation and cytokine secretion (Figure S4H–J, Supporting Information), indicating their distinct roles in inflammatory progression.

**Figure 10:**
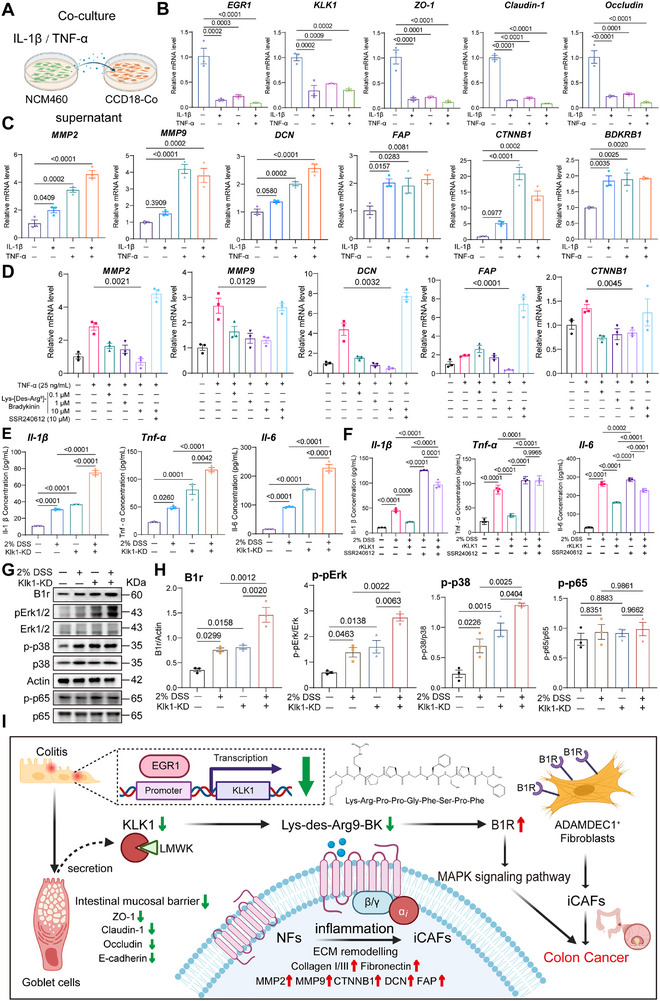
Role of Bradykinin B1 Receptor in Fibroblast Phenotypic Transition and Inflammatory Progression in Colitis. A) Schematic diagram of the experimental design for co‐culturing the supernatant of NCM460 induced by inflammatory factors with CCD‐18Co colonic fibroblasts. B) Relative mRNA levels of KLK1 and epithelial mucosal barrier genes in NCM460 cells treated with Inflammatory factors. C) After co‐culture of NCM460 supernatant induced by inflammatory factors with CCD‐18Co colonic fibroblasts, the mRNA levels of MMP pathway‐related indicators in CCD‐18Co cells were detected by qPCR. D) Relative mRNA expression of markers related to the ECM pathway and fibroblast phenotype transition (n = 3 per group). E,F) ELISA detection of Il‐1β, Tnf‐α, and Il‐6 levels in mouse serum in the KLK1‐AAV combined with 2% DSS model (E) and the SSR240612 inhibitor model (F) (n = 3 per group). G,H) Western blot detection of key phosphorylated proteins of MAPK and NF‐κB pathways in the KLK1‐AAV combined with 2% DSS model, and quantification of B1r, p‐pErk, p‐p38, and p‐p65 protein levels (n = 3 per group). I) Activation of the KLK1‐[Lys‐des‐Arg^9^‐BK]‐B1R axis under colitis promotes the occurrence of colorectal cancer by activating the MAPK pathway and transforming the phenotypic changes of fibroblasts. All data are shown as the mean ± SEM. Data are representative of three independent experiments. The *P* value was analyzed by one‐way ANOVA with Tukey's multiple comparisons, and all *P* values are marked with specific values in the graph.

We stimulated colonic epithelial cells NCM460 with inflammatory cytokines and collected the supernatants for co‐culture with colon fibroblast CCD‐18Co (**Figure** **10**A). We explored the expression of EGR1 under cytokine stimulation conditions. We stimulated with 5 ng ml^−1^ IL‐1β and 25 ng ml^−1^ TNF‐α for 24 h, and EGR1 expression decreased under cytokine‐stimulated conditions. Following inflammatory stimulation, the mRNA expression of KLK1 and mucosal barrier‐related genes in NCM460 cells significantly decreased (Figure 10B). After co‐culture, under inflammatory stimulation, CCD‐18Co fibroblasts underwent a fibroblast phenotypic transition, with increased expression of iCAFs and CAF marker genes (DCN, FAP), ECM pathway molecules, and BDKRB1 (Figure 10C).

Lys‐des‐Arg^9^‐BK is a metabolite of bradykinin and a biologically active compound formed after metabolic degradation of bradykinin. In the previous results, we demonstrated a decrease in the expression of KLK1, which is considered one of the key enzymes involved in local inflammation, and Lys‐des‐Arg^9^‐BK, formed by proteolytic cleavage of bradykinin, which acts through the B1R, in acute and chronic colitis,^[^ ^31^ ^]^ the effects of bradykinin are short‐lived in the body as it is rapidly hydrolyzed and degraded by enzymes such as bradykininase. The metabolites after bradykinin degradation include Lys‐des‐Arg^9^‐BK and des‐Arg^9^‐BK, which still have certain biological activities, especially Lys‐des‐Arg^9^‐BK, which has a strong affinity for B1R and can play an important role in chronic inflammation. To validate the role of Lys‐des‐Arg^9^‐BK binding to B1R, we co‐cultured TNF‐α‐treated NCM460 supernatants with CCD‐18Co and added varying concentrations of Lys‐des‐Arg^9^‐BK. As the concentration increased, the expression of ECM‐related genes and iCAF/CAF markers was downregulated, an effect inhibited by SSR240612 (Figure 10D).

The above results show that after KLK1 is reduced under inflammatory conditions, B1R levels are significantly increased. The results of in vitro cell experiments show that KLK1‐B1R signaling is effectively destroyed. In order to further verify the changes in functional downstream indicators after the interruption of the KLK1 signaling axis conduction, we detected Il‐1β, Tnf‐α, and Il‐6 cytokines in the in vivo animal model AAV combined with 2% DSS model. After reducing endogenous KLK1 by AAV, the level of inflammation in mice increased, and the level of inflammation in mice superimposed with 2% DSS increased significantly on this basis (Figure 10E). In the B1R inhibitor SSR240612 model, the level of inflammation in mice increased significantly under 2% DSS compared with WT, and the level of inflammation decreased significantly after supplementing KLK1 recombinant protein. When the SSR240612 group (B1R inhibitor) was given, that is, the signal transduction of KLK1 was blocked, the level of inflammation in the mice increased significantly, and the supplementation of KLK1 recombinant protein on this basis did not significantly reduce the level of inflammation. The above research results consistently showed that after KLK1 knockdown, the expression of inflammatory cytokines in colon tissue was significantly increased, and the inflammatory response downstream of KLK1 was enhanced. These results confirmed that the inflammatory signal mediated by AAV‐KLK1 was significantly enhanced, and the signal transduction of the KLK1‐B1R axis was disrupted after the administration of SSR240612 (Figure 10F).

Existing studies have shown that bradykinin can induce AQP 4 expression and the migration and invasion of glioblastoma through B1R‐mediated calcium influx and subsequent activation of the MEK1‐ERK 1/2‐NF‐κB pathway.^[^ ^32^ ^]^ In addition, B1R activation can also lead to Src phosphorylation and ADAM17 activation, which in turn leads to the activation of the downstream MAPK (ERK1/2, p38)‐PI3K/Akt signaling pathway, resulting in the secretion of MMP‐2/9 by human keratinocytes.^[^ ^33^ ^]^ We detected key phosphorylated proteins in the MAPK/NF‐κB pathway, including p‐pERK1/2, p‐p38, and p‐p65. The results showed that in the AAV2‐KLK1 knockdown model, we observed that B1R‐mediated ERK1/2 and p38 phosphorylation was significantly enhanced after KLK1 knockdown, but NF‐κB p65 phosphorylation did not change significantly (Figure 10G,H), suggesting that B1R as a GPCR may directly activate Ras‐MAPK through Gβγ‐Src in this model, rather than the typical IKK‐NF‐κB pathway. This phenomenon is similar to a previous study of sepsis. Endothelial cell B1R stimulation under inflammatory conditions leads to higher and longer‐term iNOS production through Gαi, Gβγ, and Src‐dependent activation of the ERK/MAP kinase pathway. Therefore, the development of B1R inhibitors is also a potential target for the treatment of sepsis.^[^ ^34^ ^]^ These results suggest that KLK1 deficiency may activate the MAPK pathway and drive the release of inflammatory factors by upregulating B1R expression, further supporting the role of B1R in amplifying inflammatory responses.

In summary, this study mainly revealed the mechanism of action of the KLK1‐[Lys‐des‐Arg^9^‐BK]‐B1R axis in colorectal cancer. EGR1 transcription is reduced during colitis, resulting in downregulation of KLK1 in goblet cells, which impairs mucosal barrier integrity and reduces Lys‐des‐Arg^9^‐BK levels, leading to upregulation of B1R in ADAMDEC1⁺ fibroblasts. Activation of B1R triggers MAPK signaling and promotes the transformation of fibroblasts into inflammatory cancer‐associated fibroblasts (iCAFs), which are characterized by extracellular matrix (ECM) remodeling and enhanced inflammatory signaling. The KLK1‐BDKRB1 axis promotes inflammation‐driven colorectal tumor formation by reprogramming the stromal microenvironment (Figure 10I).

## 3 Discussion

In this study, we detected the expression of KLK1 in serum and tissues of patients with ulcerative colitis, adenoma, and colorectal cancer. We found that the expression of KLK1 gradually decreased with the progression of the disease. In addition, in the DSS‐induced colitis model of mice, the expression of KLK1 in goblet cells of colitis tissue was significantly reduced, indicating that KLK1 has the functions of maintaining and repairing the intestinal mucosal barrier, regulating inflammation, reducing mucosal damage, and promoting tissue repair. It is a potential therapeutic target for UC and is expected to alleviate UC symptoms and prevent chronic inflammation from progressing to cancer. In addition, we elucidated the specific molecular mechanism of KLK1 downregulation, which may be regulated by the transcription factor EGR1. In two classic animal models of colorectal cancer, the AOM‐DSS‐induced inflammation‐cancer transformation model and the high‐fat diet‐induced *Apc* ^min/+^ adenoma carcinogenesis model, KLK1 expression was significantly downregulated. Our results indicate that the KLK1‐B1R axis activates the downstream ECM pathway and promotes the transformation of fibroblasts into iCAFs. In addition, Lys‐des‐Arg^9^‐BK can effectively repair the epithelial mucosal barrier damage caused by colitis, maintain intestinal homeostasis, and reduce the incidence of colitis‐related colon cancer and APC‐deficient adenoma carcinoma by binding to B1R.

Current studies have shown that during the progression of human IBD, goblet cells that mainly store and secrete KLK1 in the intestine are depleted and damaged, causing KLK1 to diffuse in the intestinal tissue. As inflammation progresses, KLK1 also shows a downward trend.^[^ ^35^ ^]^ Bradykinin (BK) is released from high molecular weight kininogen (HMWK) with the help of plasma kallikrein (KLK1), while lysyl bradykinin (Lys‐BK) is released from low molecular weight kininogen (LMWK) by tissue kallikrein.^[^ ^5^ ^]^ As an important component of KKS, bradykinin is mainly inactivated through angiotensin converting enzyme (ACE), which can prevent the overactivation of BK and Lys‐BK.^[^ ^36^ ^]^ Lys‐BK removes the C‐terminal arginine under the action of carboxypeptidase to become Lys‐des‐Arg⁹‐BK, and SSR240612 used in this study, is a good non‐competitive inhibitor of Lys‐des‐Arg^9^‐BK.^[^ ^37^ ^]^ At present, the research on Lys‐des‐Arg⁹‐BK mainly focuses on postoperative pain, and its antagonist SSR240612 can effectively relieve pain.^[^ ^38^ ^]^ The role of Lys‐des‐Arg⁹‐BK and its antagonists in enteritis and inflammatory‐cancer transformation is still unclear, and our work provides a new perspective.

There is still controversy over the role of B1R in the treatment of IBD. There are also reports that B1R deficiency increases the susceptibility to DSS‐induced colitis and increases the production of cytokines in DSS‐induced colitis.^[^ ^39^ ^]^ Pharmacological blockade of B1R did not alter the severity of DSS‐induced colitis, and B1R antagonists had no anti‐inflammatory or pro‐inflammatory effects on DSS‐induced colitis in mice, indicating that pharmacological blockade of B1R was insufficient to reduce the severity of DSS‐induced colitis in mice. Moreover, the absence of B1R led to intestinal epithelial barrier dysfunction and increased intestinal mucosal permeability. In certain inflammatory states, B1R may be rapidly upregulated,^[^ ^40^ ^]^ the lack of B1R increases the expression of B2 receptor mRNA in DSS‐induced colitis, so when B2 receptors are inhibited or deficient, B1R are upregulated and present some hemodynamic properties of B2 receptors, indicating the existence of a certain compensatory mechanism.^[^ ^41^ ^]^ Moreover, the loss of B1R induces compensatory upregulation of B2 receptors, and this effect is greatly exacerbated after DSS‐induced colitis, indicating that the exacerbation of colitis in mice lacking B1R is closely related to the upregulation of B2 receptors.^[^ ^39^ ^]^

KLK1 is currently mainly used in stroke. A recombinant KLK1 (rKLK1) is allowed to be used 48 h after the onset of stroke, with a dose of 0.15 U/d, intravenously injected once a day, which can effectively improve blood flow.^[^ ^42^ ^]^ Here we believe that to treat IBD patients and prevent chronic enteritis from turning into colorectal cancer, intravenous injection in the abdomen is a better method. Because KLK1 is a protease, its essence is protein, and it is not suitable for oral administration. It can reach the intestinal part quickly with high bioavailability to exert its effects. As for the dosage, further discussion is needed. In terms of the applicability of rKLK1 to human patients, it has been marketed as a stroke drug (National Medicine Standard H20052065). Its safety does not need to be evaluated too much, and its specific indications for enteritis require more clinical guidance.

Although this study revealed the key role of the KLK1‐B1R axis in intestinal barrier protection and inflammatory‐cancer transformation, there are still several limitations: 1) The controversy over the function of B1R has not been fully resolved. Existing data suggest that B1R deficiency may exacerbate inflammation through compensatory upregulation of B2 receptors, and the inhibitor experiments in this study failed to completely rule out the influence of this compensatory mechanism; 2) Although the therapeutic potential of KLK1 has been verified in animal models, its efficacy differences in human IBD patients (such as individualized response) still need to be further evaluated in preclinical trials; 3) The causal relationship between the KLK1 regulatory network (such as EGR1 transcriptional regulation) and downstream pathways (ECM remodeling, iCAFs transformation) requires more functional experimental verification. These limitations suggest that in the future, it is necessary to combine organoid models, humanized mice, and clinical cohort studies to deepen mechanism exploration and promote translational applications.

Exploring the therapeutic potential of KLK1 in inflammatory bowel disease (IBD) and developing KLK1‐related drugs is an innovative direction. Studying the expression profile of KLK1 is of great significance for personalized treatment, because different patients may have different responses to KLK1, which may bring better treatment effects to some IBD patients. Therefore, these findings deepen our understanding of the role of KLK1 in colitis and colorectal cancer and lay the foundation for new treatment strategies. KLK1 has potential application prospects as a drug for the treatment of colitis and inflammation‐related cancers or adenoma carcinoma, and needs further in‐depth research and improvement.

## 4 Experimental Section

### Animal Studies

C57BL/6 mice and C57BL/6J‐ *Apc* ^min/+^ mice aged 8 weeks were purchased from GemPharmatech Co. Ltd (Nanjing, China). The mice were raised in a stable and comfortable environment at a constant temperature of 24 °C, and provided with free access to water and standard feed. The cages were randomly divided into groups.

For acute colitis and chronic colitis induced by DSS in mice: 8‐week‐old C57BL/6 mice were randomly divided into four groups with 5 mice in each group. Model mice were given 2% DSS to drink freely for seven days, and the control group was fed with water; moreover, the other two groups of mice were respectively injected with rKLK1 and 5‐ASA. After seven days, DSS was replaced with water, and the mice were killed two days later for samples. The chronic colitis model was similar to the above method. 2% DSS was given for three cycles. Water was given after each cycle to recover for 2 weeks, and water was given after the last cycle to recover for 3 weeks. The weight, feces, and blood in the stool of the mice were recorded every day, and the disease activity index (DAI) was calculated. The colon of the mice was taken, the length was recorded, and photographed.

For the induction of AOM‐DSS model in mice: 8‐week‐old C57BL/6 mice were randomly divided into four groups, with 5 mice in each group: normal group, model group, and rKLK1 treatment group and SSR240612 administration group were given on the basis of the model group. Azomethane (AOM, 7.5 mg kg^−1^) was injected intraperitoneally after the mice had adapted to the environment, and then they were fed normally for one week. On the 7th day, the drinking water of the model group and the drug group was replaced with water containing 2% DSS. The weight of the mice was recorded every two days. After one week, it was replaced with normal drinking water and continued to be fed for two weeks. DSS drinking water was given twice more, and feeding was continued for 4 weeks after the third DSS. The intestines of the mice were examined on the 80th day to observe the colon length and tumors.

To establish a murine model with Intestinal epithelial cells‐specific Klk1 knockdown, adeno‐associated virus AAV2 (serotype 2) delivered via enema injection was employed. Two recombinant AAV2 vectors were constructed: AAV2‐NC (negative control) and AAV2‐Klk1 (pAAV‐U6‐shRNA (NC/Klk1)‐CMV‐EGFP‐WPRE). Eight‐week‐old mice were administered the AAV2 virus by enema before drinking 2% DSS. After three weeks of virus proliferation and expression in the mice, acute colitis was induced by DSS. Each experimental group consisted of 5 mice. The shRNA sequences were as follows: shRNA‐NC: TTCTCCGAACGTGTCACGT; shRNA‐Klk1: CATGTTGTGTGCAGGAGAT.

In the *Apc* ^min/+^ mouse model of colorectal cancer precancerous lesions, *Apc* ^min/+^ mice were randomly allocated into four experimental groups following an adaptation period: wild‐type control group, model control group (*Apc* ^min/+^), rKLK1 supplementation group, and SSR240612 treatment group, with five mice in each group. Concurrently with group assignment, the standard diet was replaced with a high‐fat diet (60% fat content). Body weight was monitored weekly throughout the 10‐week modeling period.

### Immunofluorescence and Immunohistochemistry

Intestinal tissues (including Swiss roll colon samples) were fixed in 4% paraformaldehyde, embedded in paraffin, and sectioned into 6‐µm slices for immunofluorescence (IF) and immunohistochemistry (IHC) staining. For IF staining, sections were permeabilized with 0.2% Triton X‐100, blocked with 5% bovine serum albumin (BSA) for 1 h, and incubated overnight at 4 °C with primary antibodies, including: MUC2 (Abclonal, Cat# A14659), BDKRB1 (Proteintech, Cat# 26672‐1‐AP), Mmp2 (Proteintech, Cat# 10373‐2‐AP/30592‐1‐AP), Dcn (Abclonal, Cat# A15048), Fap (Abcam, Cat# AB218164), Fibronectin (Affinity Bioscience, Cat# AF5335), Collagen I (Affinity Bioscience, Cat# AF7001), Collagen III (Affinity Bioscience, Cat# AF5457), KLK1 (SAB, Cat# 32 443), β‐Catenin (CST, Cat# 8480S), eGFP (Invitrogen, Cat# CAB4211), Tight Junction Protein 1 (Proteintech, Cat# 66452‐1‐Ig), E‐cadherin (CST, Cat# 3195S), and Occludin (Proteintech, Cat# 27260‐1‐AP). After washing, sections were incubated with anti‐rabbit Alexa Fluor 488 (Invitrogen) or anti‐mouse Alexa Fluor 568 (1:500 dilution) secondary antibodies for 1 h at room temperature, followed by treatment with HRP‐conjugated secondary antibodies and Tyramide Signal Amplification kits. For IHC staining, the procedure was similar to IF staining: after antigen retrieval, sections were washed and incubated with isotype‐specific HRP‐conjugated anti‐mouse or anti‐rabbit secondary antibodies. Finally, images were acquired using an Olympus BX51 fluorescence microscope. The quantitative analysis of the image was accomplished by ImageJ software.

### Drug and Solvent Dissolution Scheme

Urinary Kallidinogenase for Injection: 0.15 PAN U (1 bottle) Urinary Kallidinogenase for Injection (Shanghai Techpool, National Medicine Standard H20052065) + 1 mL of normal saline as storage solution; 1.5×10^−3^ PAN U kg^−1^ was injected intraperitoneally into rats. The simple calculation scheme was to inject 100 µL of Urinary Kallidinogenase for Injection per 20 g body weight. The drug Urinary Kallidinogenase for Injection has been used clinically. The active ingredient was KLK1 extracted from human urine, which plays a pharmacological role in cleaving kininogen to produce bradykinin.^[^ ^43^ ^]^ Since the cost of purchasing recombinant KLK1 protein for animals was too high, and the pharmacokinetics of drugs injected into animals without excipients may also be affected, and the finished drug has many mature application scenarios and stable pharmacokinetics, this part of the study selected Urinary Kallidinogenase for Injection to replace recombinant KLK1.

5‐Amino Salicylic Acid was dissolved in saline and administered by oral gavage 100 µL at a dose of 100 mg kg^−1^; Icatibant/HOE‐140 (MCE, Cat# HY‐17446): dissolved in 5% TW80 and injected intraperitoneally at 3 mg kg^−1^, 100 µL per mouse; SSR240612 (GLPBIO, Cat# GC19339): dissolved in 5% TW80 and injected intraperitoneally at 1 mg kg^−1^, 100 µL per mouse. SSR240612 (MCE, Cat# HY‐15039) and Lys‐[Des‐Arg^9^]‐Bradykinin TFA (MCE, Cat# HY‐103295A) as well as Recombinant Protein of IL1β (PeproTech, Cat# 211‐11B) and TNF‐α (PeproTech, Cat# 315‐01A) were used for cell experiments.

### Single‐Cell Sequencing Data Processing

The 10× Chromium single‐cell gene expression data were processed using the companion software CellRanger (v3.1.0) for alignment, barcode assignment, and unique molecular identifier (UMI) counting (using the reference set GRCh38‐3.0.0). Filtered count matrices were converted to sparse matrices using the Seurat package (v4.3.0)^[^ ^44^ ^]^ in R. To avoid batch effects among samples and experiments, integration of single‐cell data was performed using the Harmony integration tool.^[^ ^45^ ^]^

### Inflammation Score

Based on the single‐cell dataset, the relative expression levels of inflammation‐related genes were calculated in each sample, using the relative expression levels of gene sets as markers to assess inflammation characteristics. The genes used to calculate inflammation characteristics include 42 inflammation‐related genes such as I*FNG*, *IFNGR1*, *IFNGR2*, *IL10*, *IL12A*, *IL12B*, *IL12RB1*, *IL12RB2*, *IL13*, *IL17A, and IL17F*. The inflammation gene set was derived from the colitis‐related inflammation genes defined by Smillie et al.^[^ ^8^ ^]^

### Enzyme‐Linked Immunosorbent Assay (ELISA)

Mouse serum KLK1 concentrations were measured by Mouse KLK1 (Kallikrein 1) ELISA Kit (ELK Biotechnology, Cat# ELK4995). Human serum KLK1 concentrations were determined by Human KLK1(Kallikrein 1) ELISA Kit (ELK Biotechnology, Cat# ELK4888). Inflammatory cytokines in mouse serum were detected by mouse Il6 (Interleukin 6) microsample ELISA Kit (ELK Biotechnology, Cat# ELK1157MS), mouse Il‐1 beta ELISA Kit (proteintech, Cat# KE10003), and mouse Tnf‐alpha ELISA Kit (proteintech, Cat# KE10002).

### ChIP Assays

ChIP assays were performed by ChIP Assay Kit (Beyotime Biotechnology, Cat# P2078) according to the instructions. ChIPbase software was used to predict transcription factors that may regulate KLK1, and ChIP‐PCR was further used to verify the transcription factor binding sites by designing primers for specific sites. The DNA binding site of transcription factor EGR1 was verified by PCR experiments using EGR1 antibody (proteintech, Cat# 22008‐1‐AP). DNA purification by using a PCR purification kit/DNA purification kit (Beyotime Biotechnology, Cat# D0033).

### DAI

The Disease Activity Index (DAI) for assessing intestinal inflammation was calculated using an established scoring system. Scores ranged from 0 to 4 based on the following criteria: weight loss (0, < 1%; 1, 1–5%; 2, 6–10%; 3, 11–18%; 4, > 18%), stool consistency (0, normal; 1, soft but formed; 2, soft; 3, very soft and moist; 4, watery diarrhea), and bleeding (0 and 1, negative hemoccult; 2, positive hemoccult; 3, visible blood traces in stool; 4, gross rectal bleeding). The final DAI score was determined by averaging the combined scores from these parameters.

### Quantitative Real‐Time PCR Analysis

The 20 µL reaction mixture was prepared by combining 2×Taq PCR Premix (KT201) with cDNA templates, forward/reverse primers, and sterile distilled water. Thermal cycling conditions were programmed as follows: initial denaturation at 94 °C for 2 min; followed by 25‐30 cycles of denaturation (94 °C, 30 sec), primer annealing (60 °C, 30 sec), and extension (72 °C, 45 sec), with a final elongation step at 72 °C for 5 min. Amplification was carried out on the GeneAmp PCR System 9700 thermal cycler. Primer sequences are provided in Table S2 (Supporting Information). Following agarose gel electrophoresis, amplification products were visualized and documented using the ChemiDoc XRS+ Imaging System.

### Western Blot

Sample preparation begins by lysing cells/tissues with RIPA buffer containing 1% protease inhibitor cocktail on ice for 30 min, followed by centrifugation at 12000×g (4 °C) for 15 min to collect supernatants. Protein concentration was quantified using BCA assay, and 20–50µg proteins were mixed with 5×SDS loading buffer, then denatured at 95 °C for 5 min. SDS‐PAGE electrophoresis was performed using the Bio‐Rad Mini‐PROTEAN system with 12% separating gel and 5% stacking gel, loading 20µL samples per lane. Electrophoresis starts at 80V for the stacking gel and switches to 120V for the separating gel, terminating when bromophenol blue reaches the gel bottom. Protein transfer was conducted via Bio‐Rad Trans‐Blot wet transfer system at 200mA for 90 min using transfer buffer (25mM Tris‐HCl, 192mM glycine, 20% methanol), with PVDF membranes pre‐activated in methanol for 1 min. Membranes were blocked with 5% non‐fat milk in TBST for 1 h at room temperature, incubated with primary antibodies (1:1000 dilution) overnight at 4 °C with shaking, washed 3×10 min with TBST, then treated with HRP‐conjugated secondary antibodies (1:5000 dilution) for 1 h at RT. Detection employs ECL chemiluminescence, and signals were captured by the ChemiDoc imaging system, followed by band intensity quantification using Image Lab software. Critical precautions include maintaining samples on ice throughout procedures, and cooling the transfer system with an ice bath.

### Histology Score

The histology scoring system evaluates colonic mucosal damage through two parameters: severity and extent of injury. For mucosal damage severity, scores were assigned as follows: 0 indicates normal colonic mucosa; 1 represents loss of one‐third crypt glands; 2 denotes loss of two‐thirds crypt glands; 3 signifies complete crypt gland loss; and 4 indicates mucosal epithelial erosion with destruction accompanied by significant inflammatory cell infiltration. For mucosal damage extent, the scoring criteria are: 0 for normal colonic mucosa; 1 for localized mucosal damage; 2 for damage involving one‐third of the intestinal area; 3 for damage covering two‐thirds of the intestinal area; and 4 for damage extending throughout the entire intestinal tract. The final histology score was calculated by summing the severity and extent scores.

### IHC Score

A score of 0 indicates no staining (negative); a score of 1 represents weak staining (light yellow coloration, observable only under high magnification); a score of 2 denotes moderate staining (brownish‐yellow coloration, clearly visible under medium magnification); and a score of 3 signifies strong staining (dark brown coloration, identifiable even under low magnification).

### FITC‐Dextran Intestinal Permeability Assay

The intestinal barrier integrity was assessed by the fluorescein isothiocyanate‐dextran tracing technique. After the DSS‐induced colitis model was completed, the mice were fasted for 12 h and given 200 µL of fluorescein isothiocyanate‐dextran solution (60, 600 mg kg^−1^ body weight) by oral gavage. Retroorbital blood was collected from the mice 4 h after administration. The level of fluorescein isothiocyanate‐dextran in serum was analyzed by a fluorescence microplate reader (excitation/emission wavelength: 485/535 nm) and quantified according to the standard curve.

### Murine Colonoscopy

The murine colonoscopy procedure was conducted using an animal electronic endoscope (Model: MiniScope 2V) for in vivo intestinal visualization. Mice were fasted for 12 h (with free access to water) preoperatively, anesthetized, and secured in a supine position on a thermostatic surgical platform (37 °C). A flexible endoscope (outer diameter: 2.2 mm) was gently inserted transanally, followed by air insufflation (1 mL) via syringe to achieve intestinal distension. Retrograde observation from the colorectum to the ileocecal junction was performed, and mucosal lesions were captured via an imaging acquisition system.

### Cell Culture

Normal human colon epithelial cells (NCM460) and Human normal colon fibroblasts CCD‐18Co) were purchased from the Chinese Academy of Medical Sciences (Beijing, China) and were characterized by short tandem repeat sequencing. NCM460 cells were cultured in RPMI 1640 medium (Gibco, Grand Island, USA) supplemented with 10% fetal bovine serum (Gibco) and CCD‐18Co cells were cultured in MEM medium (Gibco, Grand Island, USA) supplemented with 10% fetal bovine serum, maintained at 37 °C and 5% CO_2_ Once the cell density reached 1 × 106 cells mL^−1^, digestion and cell passaging were performed using 0.1% trypsin solution (Gibco). Typically, the passaging ratio is 1:2 to 1:3 every 2–3 days to ensure that the cells remain in the logarithmic growth phase and to avoid excessive proliferation. There was no reason to be infected with Mycoplasma throughout the experiment.

### Data Availability

The AOM‐DSS animal model scRNA‐seq data have been deposited to the GEO database with accession number GSE264607, and *Apc* ^min/+^ animal model accession number was GSE277268. Human ulcerative colitis data were obtained by integrating SCP259^8^ and the own dataset GSE168025. The RNA‐sequencing data can be accessed on the Gene Expression Omnibus repository using the accession number GSE282383.

### Statistics

GraphPad Prism software was used for statistical evaluation (GraphPad, Inc., Chicago, IL, USA). The data were expressed as the mean ± SEM of at least three independent experiments. For parametric comparison, one‐way analysis of variance (ANOVA) followed by Tukey's test for multiple groups, or a two‐tailed, unpaired Student's t‐test for two groups, was used. For nonparametric comparison, a Mann‐Whitney test was used. *p* < 0.05 was considered statistically significant. Statistical analysis was conducted using GraphPad Prism 9.4.0 software.

## Conflict of Interest

The authors declare no conflict of interest.

## Author Contributors

L.Z., M.W., S.L., and L.G. contributed equally to this work. Y.S., H.C., H.C., and T.L. designed the study. L.Z., M.W., S.L., T.Z., and L.Z. performed the experiments. L.Z. and Y.M. analyzed the data. H.C., H.C., L.G., T.L., Q.D., S.L., and J.W. collected of clinical samples. L.Z. and Y.S. wrote the manuscript. All authors reviewed and approved the manuscript.

## Ethics Approval

The human study was approved by the Research and Ethical Committee of Affiliated Hospital of Nanjing University of Chinese Medicine (2021‐NL‐068‐02) and the Ethical Committee of Shanghai Sixth People's Hospital (2022‐KY‐124K). Informed consent was obtained from all participants. Mice were purchased from GemPharmatech Co. Ltd (Nanjing, China). All animal experiments were conducted in accordance with the National Institutes of Health Guide for the Care and Use of Laboratory Animals and were approved by the Experimental Animal Care and Use Committee of Nanjing University (no. IACUC‐2401006 and IACUC‐2502014).

## Supporting information



Supporting Information

Supplemental Table 1

Supplemental Table 2

Supplemental Table 3

Supplemental Table 4

## Data Availability

The data that support the findings of this study are available in the supplementary material of this article.
