# Supplementary material for: KLK1 as an Epithelial‐Specific Brake Inhibits Colorectal Tumorigenesis by Suppressing B1R‐Mediated Fibroblast Phenotypic Transition
Source: Adv Sci (Weinh). 2025 Aug 26;12(42):e07063. doi: 10.1002/advs.202507063 (PMC12622486; doi:10.1002/advs.202507063)
Supplement: Supplementary file 1 — Supporting Information [file ADVS-12-e07063-s005.docx]

**Supplementary figure 1**


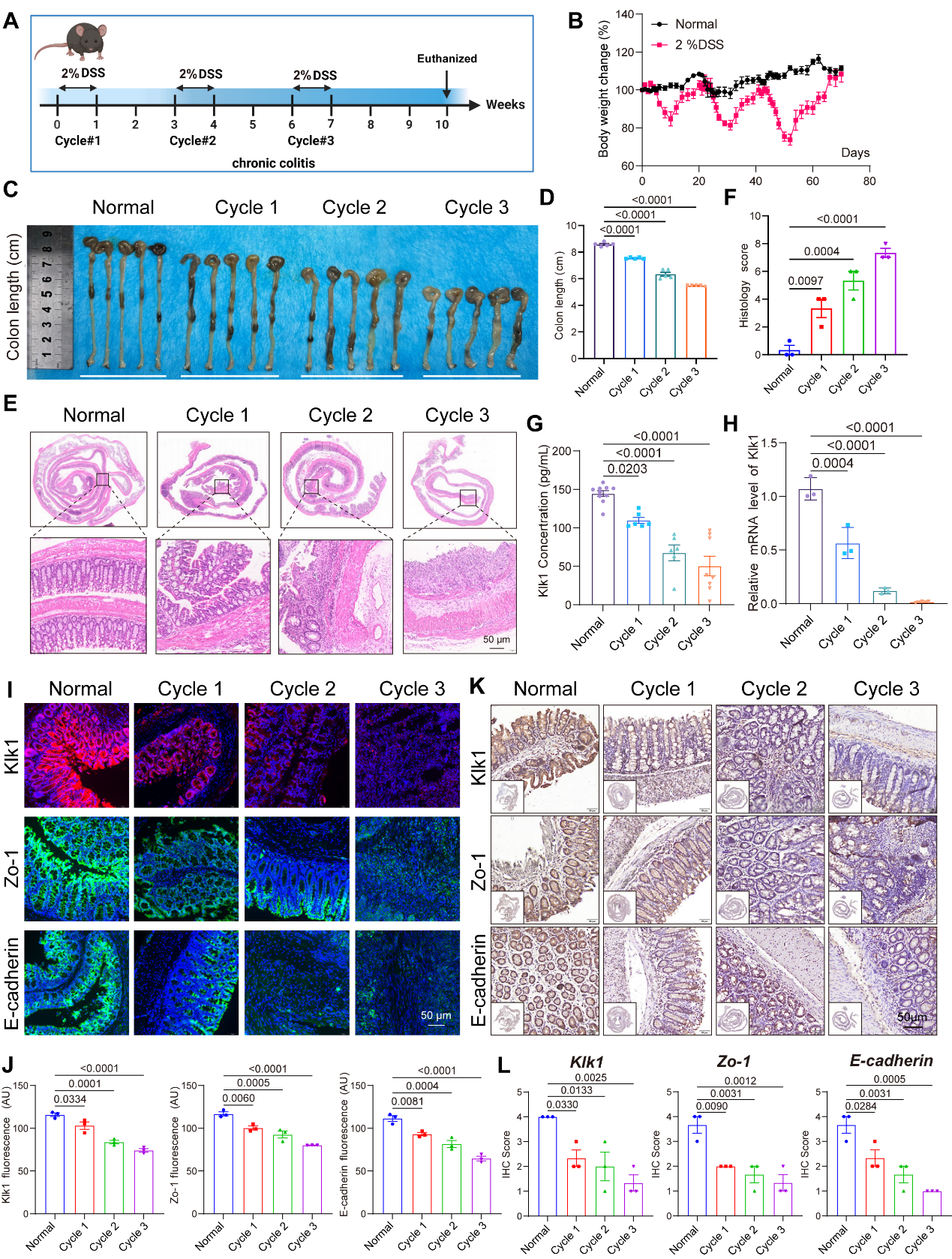


**Supplementary figure 1. KLK1 is decreased in DSS-induced chronic colitis model mice.**

(**A**) Schematic diagram of the experimental design of 2% DSS-induced chronic colitis. (**B**) Body weight curves denote the changes in the mean body weight of mice recorded daily in different groups.

(**C**) Representative picture of the colons from different groups and (**D**) colon length (n=5 per group).

(**E**) The histopathological grading of inflammation in C57BL/6 mice colon with DSS-induced chronic colitis.

(**F**) Photomicrographs show representative images of H&E staining (n=3 per group).

(**G**) Detection of mouse serum Klk1 concentration by ELISA (n>5 per group).

(**H**) Relative mRNA level of Klk1 in 2% DSS-induced chronic colitis.

(**I-J**) Immunofluorescence staining of Klk1, Zo-1 and E-cadherin in C57BL/6 mice colon with Klk1 (red), Zo-1 (green), E-cadherin (green) and DAPI (blue) antibodies (n=3 per group).

(**K-L**) Immunohistochemistry of Swiss rolls showing the changes in the expression of Klk1, Zo-1 and E-cadherin in chronic colitis model and (**L**) Data are representative of three independent experiments.

Scale bars: 50 μm. All data are shown as the mean ± SEM. Data are representative of three independent experiments. The *P* value was analyzed by one-way ANOVA with Tukey's multiple comparisons and all *P* values are marked with specific values in the graph.

**Supplementary figure 2**


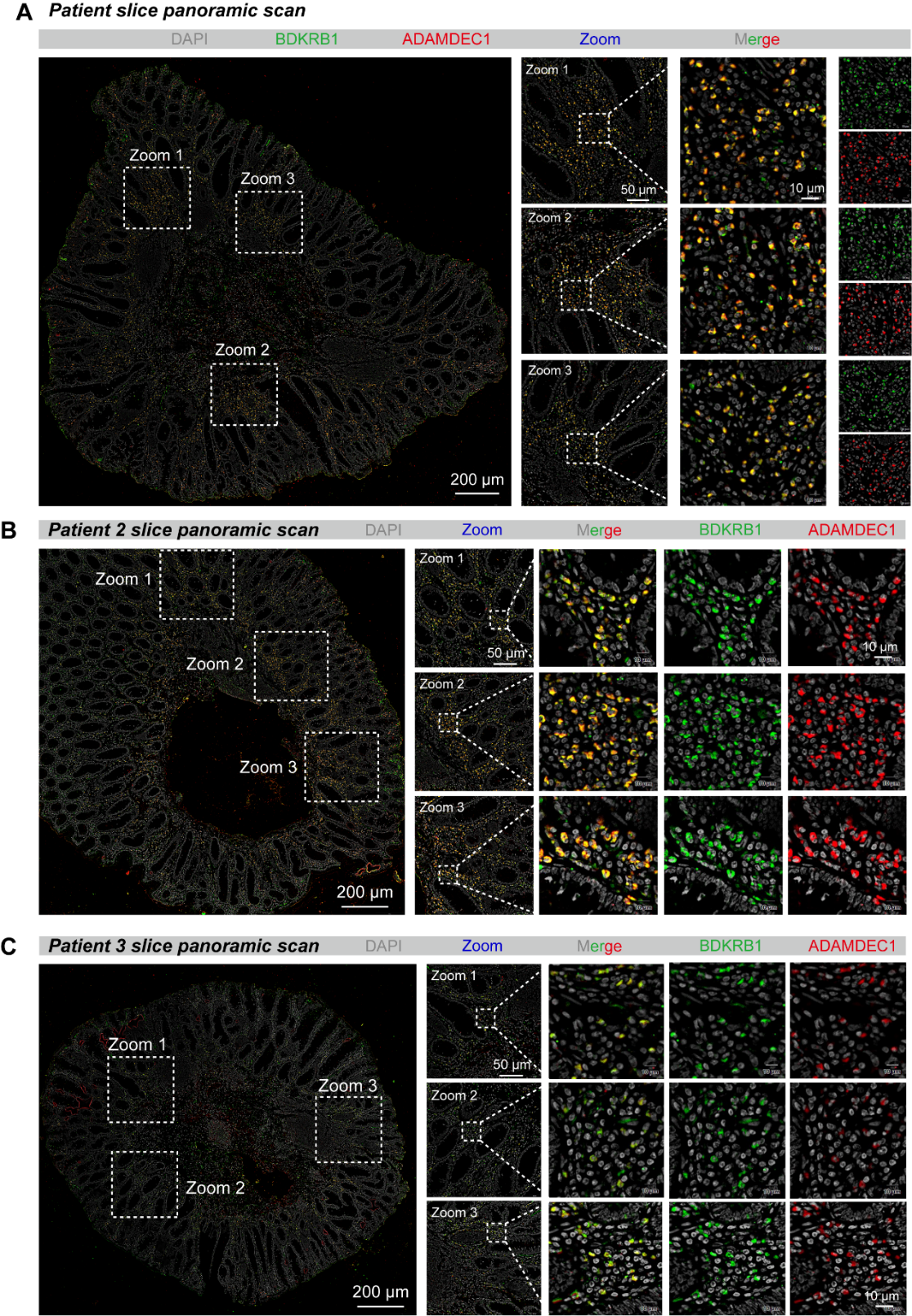


**Supplementary figure 2. B1R is induced by inflammation and is highly expressed in ADAMDEC1^+^ fibroblasts**

(**A**-**C**) Immunofluorescence staining of BDKRB1^+^ ADAMDEC1^+^ cells in patient with colorectal adenoma, BDKRB1 (green), ADAMDEC1 (red), and DAPI (grey) antibodies. Data are representative of three independent experiments. Patient information is provided in Supplementary Table 4 (Supporting Information). BDKRB1 colocalizes with ADAMDEC1^+^ fibroblasts in patients with colorectal adenoma. Scale bars: 200 μm. Zoom area scale bars: 10μm.

**
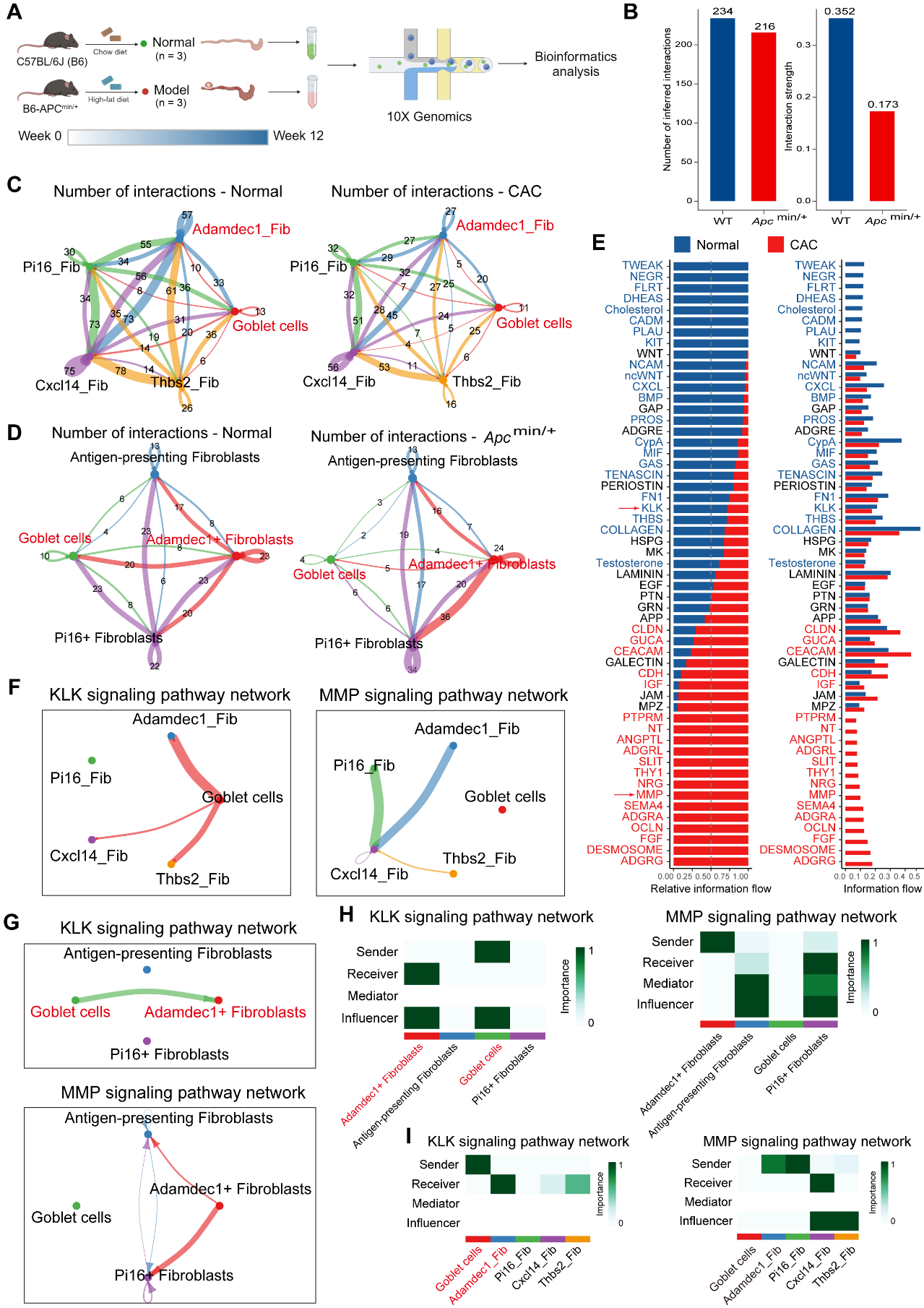
Supplementary figure 3**

**Supplementary figure 3.** **Cellular communication in the AOM-DSS induced inflammation-cancer transformation model and the *Apc*^min/+^ adenoma carcinoma scRNA-seq dataset.**

(**A**) The schematic diagram of sample collection, scRNA-seq transcriptomic analysis of the *Apc*^min/+^ adenoma carcinoma mode.

(**B**) The bar chat showing the total number of interactions and interaction strength of the inferred cell-cell communication networks from different biological conditions between normal and *Apc*^min/+^ adenoma carcinoma.

(**C**) Circle plot showing the number of interactions between normal and AOM-DSS induced inflammation-cancer transformation model in BDKRB1^+^ fibroblast subtypes. (**D**) Circle plot showing the number of interactions between normal and *Apc*^min/+^ in BDKRB1^+^ fibroblast subtypes.

(**E**) Bar chart showing the Significant signaling pathways which ranked based on differences in the overall information flow within the inferred networks between Normal and AOM-DSS induced adenoma.

(**F**) The Chord diagram visually compare cell-cell communication in KLK and MMP signaling pathways in the AOM-DSS induced inflammation-cancer transformation model.

(**G**) The Chord diagram visually compare cell-cell communication in KLK and MMP signaling pathways in the *Apc*^min/+^ adenoma carcinoma.

(**H**) Calculate the network centrality measure for each cell group of AOM-DSS induced inflammation-cancer transformation model to identify the main senders, receivers, mediators, and influencers in the intercellular communication network and their contributions to the KLK and MMP signaling pathways.

(**I**) Calculate the network centrality measure for each cell group of *Apc*^min/+^ adenoma carcinoma model to identify the main senders, receivers, mediators, and influencers in the intercellular communication network and their contributions to the KLK and MMP signaling pathways.


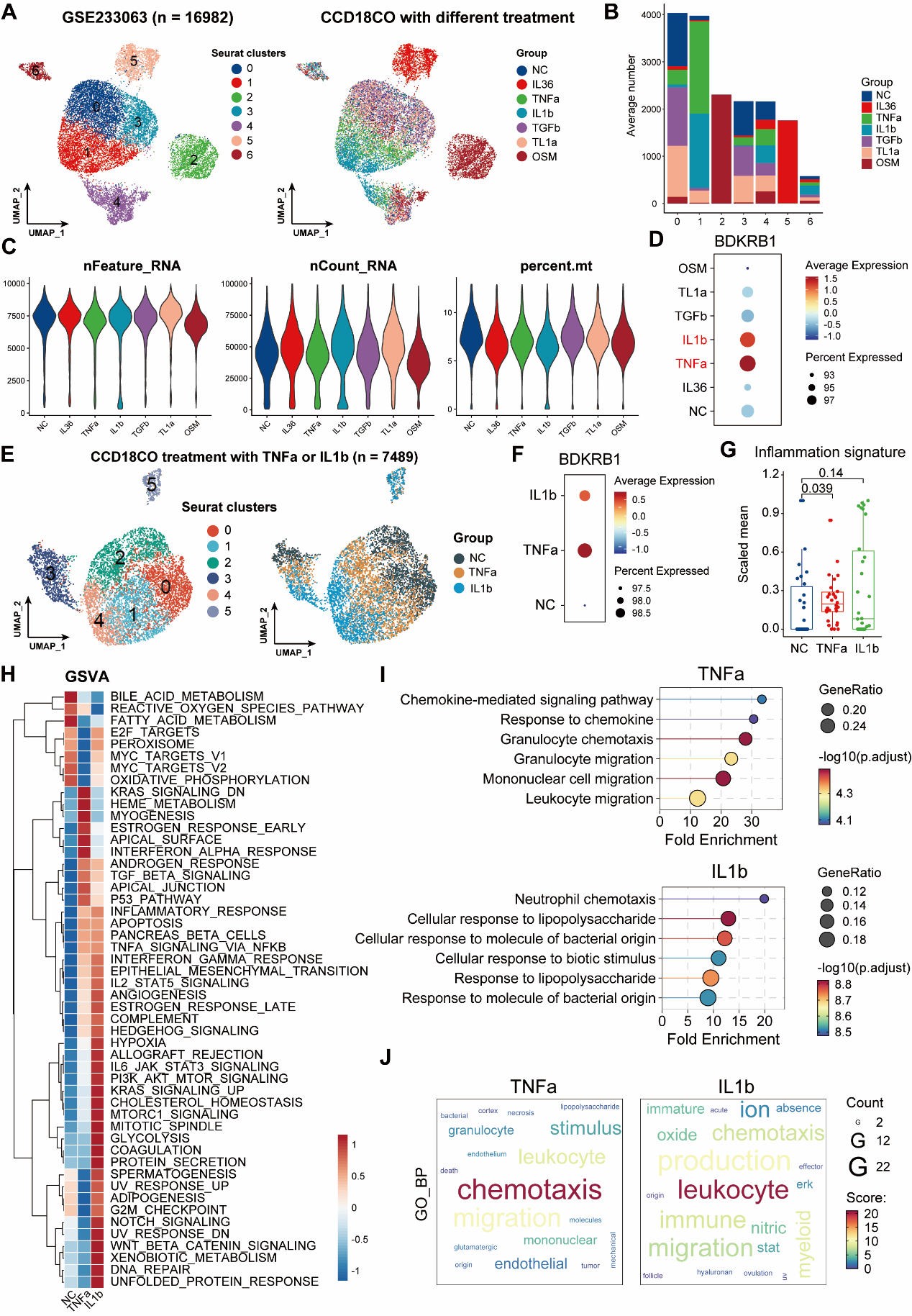
**Supplementary figure 4**

**Supplementary figure 4. CCD18co colonic fibroblast cell line treated with various pro-inflammatory stimuli and analyzed by scRNA-seq.**

(**A**) UMAP of 16982 cells analyzed by scRNA-seq across 7 groups.

(**B**) Bar plot shows the average number of cells in different groups.

(**C**) Violin plot showing the quality control of GSE233063: we filtered cells that have unique feature counts over 10000 or less than 200 and filtered cells that have >15% mitochondrial counts.

(**D**) Dot plot showing the expression levels of BDKRB1 in various pro-inflammatory stimuli groups. Dot size indicates the fraction of expressing cells and the colors represent normalized gene expression levels.

(**E**) UMAP of 7489 cells which treated with TNF-α and IL1β.

(**F**) Dot plot showing the expression levels of BDKRB1 in TNF-αand IL1β stimuli groups. Dot size indicates the fraction of expressing cells and the colors represent normalized gene expression levels.

(**G**) Box plot showing the mean expression of inflammation signatures in TNF-α and IL1β stimuli groups. The boxes indicate the 25% quantile, median, and 75% quantile; the points indicate the individual signatures.

(**H**) Heatmaps showing HALLMARK pathways enriched in TNF-α and IL1β stimuli groups by using GSVA analysis.

(**I**) Lollipop diagram showing the enriched pathways in TNF-α and IL1β stimuli groups. (**J**) Word cloud map displaying the most significant signatures in TNF-α and IL1β stimuli groups by using GO enrichment analysis.
